# Regenerable Graphene Nanoplatelet Adsorbents for Rapid and Trace-Level PFAS Removal from Water

**DOI:** 10.1021/acsomega.5c08997

**Published:** 2026-01-05

**Authors:** Md. Nahid Pervez, Tao Jiang, Aswin Kumar Ilango, Yamini Kumaran, Yi Zhang, Weilan Zhang, Haralabos Efstathiadis, Jeremy I. Feldblyum, Mehmet V. Yigit, Arjun K. Venkatesan, Yanna Liang

**Affiliations:** † Department of Environmental and Sustainable Engineering, University at Albany, State University of New York, Albany, New York 12222, United States; ‡ Department of Nanoscale Science and Engineering, University at Albany, State University of New York, Albany, New York 12222, United States; § New York State Center for Clean Water Technology, 12301 Stony Brook University, Stony Brook, New York 11794, United States; ∥ Department of Chemistry, University at Albany, State University of New York, Albany, New York 12222, United States; ⊥ Department of Civil and Environmental Engineering, 5965 New Jersey Institute of Technology, Newark, New Jersey 07102, United States

## Abstract

Per- and polyfluoroalkyl substances (PFAS) are persistent synthetic chemicals of global concern, and adsorption remains one of the most efficient methods for their removal from water. The present research reported a new kind of graphene nanoplatelets (GNP) modified by a cationic surfactant, cetyltrimethylammonium chloride (CTAC). This GNP-CTAC was observed to achieve nearly 100% capture of all 10 target PFAS, each at 10 μg/L in Milli-Q water. The pseudo-second-order model most accurately represented the adsorption kinetics, which was realized as a fast adsorption process in less than 1 min. The Sips isotherm model effectively fits the isotherm data, indicating that the adsorption of PFAS onto GNP-CTAC involved both heterogeneous surface sites and multilayer adsorption driven by combined electrostatic and hydrophobic interactions. The hypothesized adsorption mechanisms, including electrostatic and hydrophobic interactions, were validated by detailed physicochemical characterization. Remarkably, the performance of GNP-CTAC remained unaffected by variations in solution pH, ionic strength, natural organic matter, and nearly 100% removal effectiveness of PFAS in river water (surpassing conventional adsorbents like powdered activated carbon) at an initial PFAS concentration of 200 ng/L. Importantly, the adsorption performance of GNP-CTAC was successfully validated through a third-party evaluation. Along with its rapid adsorption of PFAS and stability across various water qualities, GNP-CTAC was also regenerable and could be reused for a minimum of four cycles, retaining most of its adsorption performance. Notably, stability evaluations confirmed that CTAC remained attached to the GNP surface during regeneration and adsorption, with no signs of surfactant leakage.

## Graphical abstract

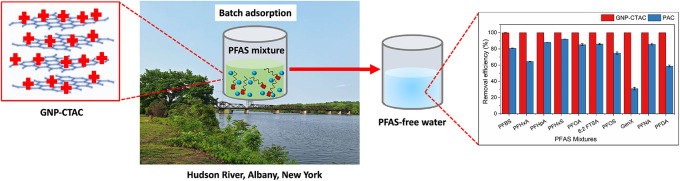

## 1 Introduction

Per- and polyfluoroalkyl substances (PFAS) are a wide range of man-made organic compounds that consist of various compounds where the hydrogen atoms connected to the carbon backbones are substituted with fluorine, either completely or partly.^1–3^ The unique attributes of PFAS have enabled their widespread use in diverse industrial processes and consumer products, including surfactants for mining and oil extraction,^4,5^ coatings for textiles and food packaging,^6,7^ foams for creating water-based films,^8,9^ cosmetics and personal care products,^10^ cleaning agents,^11^ and various other applications.^12^ PFAS, which have been manufactured for over 60 years and are now extensively detected in the ecosystem, are causing grave concerns owing to their potential hazards to human health and the environment.^13,14^ Therefore, it is crucial to promptly and urgently take measures to remove PFAS from contaminated environments.

In this regard, a number of methods, including, but not limited to adsorption/filtration, chemical/electrochemical destruction, and biological degradation, have been explored.^15–17^ Membrane filtration can effectively remove PFAS from water but often require high-pressure systems and generate secondary waste.^18^ Chemical/electrochemical destruction can degrade PFAS to certain degrees, yet they are generally energy-intensive, costly, and may produce toxic byproducts.^19^ Biological degradation are environmentally friendly but typically slow and inefficient for persistent PFAS.^20^ Among numerous remediation technologies, adsorption has emerged as a promising remediation approach because it combines high removal efficiency with operational simplicity, user-friendliness, and cost-effectiveness.^21,22^ Countless adsorbents have been developed for PFAS removal, such as carbon-based materials,^23,24^ ion-exchange resins,^25^ biosorbents,^26,27^ and clay-based materials.^28,29^ The most prominent carbon-based materials used for PFAS adsorption are activated carbon (AC), which include granular and powdered AC (GAC and PAC), carbon nanotubes (CNTs), and biochar.^30,31^

Graphene-based carbon materials have been continuously explored as high-performance adsorbents for PFAS capture.^24,32,33^ Graphene oxide (GO) is the most prevalent derivative of graphene, characterized by a multitude of oxygen-containing functional groups such as carbonyl, carboxyl, and hydroxyl. As a result, GO has been used to capture PFAS from water through hydrophobic and electrostatic interactions.^34^ For example, polyethylenimine-functionalized GO has been shown to be effective in the removal of perfluorooctanoic acid (PFOA) from water, in which the surface structure was the primary driving force behind the high efficiency.^35^ Amino-functionalized GO aerogel also enhanced the removal of PFOA due to the formation of interconnecting porosity microstructures and the presence of amino groups.^36^ The GO-based materials’ tunable layered structure, surface area, chemical stability, and reactivity are key factors for their effectiveness in PFAS removal. Despite these benefits, GO materials still face major obstacles, such as poor adsorption of highly hydrophilic short-chain PFAS, postadsorption separation problems, and cost. Because of these challenges, improved adsorbents made of graphene derivatives that are both stable and inexpensive are desired for PFAS cleanup.

Graphene nanoplatelets (GNPs)-derived materials that are chemically and morphologically comparable to ideal graphene have seen a dramatic increase in production in recent years, suggesting that they may be the key to solving these problems.^37^ Aromatic carbon–carbon rings make up the bulk of GNP sheets, which normally have many layers in the basal plane. Intercalation and exfoliation damage the conjugated structure, making the margins of these rings oxidation-prone. GNP has many advantages over other materials, including the ability to self-template, being very light, and having a high aspect ratio.^38,39^ Their surface structure may be modified to enhance PFAS adsorption. Additionally, GNPs could be engineered to include functional components because of their diminutive size and form. This makes them amenable to chemical modification and dispersion, allowing them to circumvent diffusion constraints that are inherent in conventional graphene-based materials. Moreover, GNP-based adsorbents have been successfully employed for the remediation of contaminants such as heavy metals (e.g., As­(V)),^40^ pharmaceuticals (e.g., aspirin, caffeine, and acetaminophen),^41,42^ and other organic pollutants (e.g., naphthalene).^43,44^ These prior evidence motivated us to explore the potential of GNP-based adsorbents for PFAS capture.

Here, we report a simplified synthesis of a novel adsorbent by modifying GNP with a cationic surfactant (cetyltrimethylammonium chloride, CTAC) and evaluate its ability to remove a mixture of PFAS from an aqueous medium. CTAC was selected as the intercalating agent from the class of cationic surfactants because such compounds possess a hydrophilic head with a positively charged quaternary ammonium group and hydrophobic alkyl chains, enabling simultaneous electrostatic attraction with anionic PFAS head groups and hydrophobic interaction with their fluorinated tails. Previous studies demonstrated that cationic surfactant-modified adsorbents are effective for anionic PFAS capture due to their positive charges.^24,28,29^ Also, the modification may effectively disperse GNP by physically attaching it with surfactant molecules without causing oxidation or defects, maintaining its integrity,^45^ lowering surface energy, increasing the availability of few-layer graphene by peeling, and preventing reaggregation.^46^ Our study thus focused on the design and synthesis of GNP-derived functional materials with two distinct characteristics: (1) carbon-rich nanoscale graphene with positive surface charge and (2) self-dispersion obtained with controlled size through surfactant modification. Specifically, this study aimed to investigate how the surfactant modification influenced PFAS adsorption comprehensively. By systematically comparing GNP and GNP-CTAC, we sought to elucidate the roles of CTAC doping, particle size, and surface charge in capturing both long- and short-chain PFAS. To our knowledge, this study represents the first exploration of this particular material specifically for PFAS adsorption. Our findings offer valuable insights into the design of next-generation graphene-based adsorbents with optimized properties such as enhanced adsorption capacity, faster kinetics, and improved reusability for PFAS remediation in real water matrices.

## 2 Experimental Section

### 2.1 Chemical Reagents and Materials

The specific information on the chemical reagents and the physicochemical characteristics of PFAS, as well as the analysis carried out in this study, can be found in Tables S1–S3, respectively. The surface water was obtained from the nearby Hudson River in Albany, NY, USA, and kept at 4 °C before use. The composition of river water is detailed in Table S4. Milli-Q water with a resistivity of at least 18.2 MΩ·cm was used for the preparation of working solutions throughout the experiment.

### 2.2 Surface Modification of Graphene Nanoplatelets

The GNP-CTAC was synthesized using the method described by Yang and Li^47^ with minor adjustments. In summary, a solution was prepared by dispersing 15 mg of GNP in 50 mL of water using ultrasonic agitation for 4 h and adding 30 mg of CTAC. The suspension was left undisturbed for 12 h, during which it naturally separated into distinct layers. After centrifuging the suspension at 4500 rpm for 15 min, the solid was collected, rinsed 2–3 times with deionized water, and subjected to freezing at −20 °C for 24 h, after which it was freeze-dried at −45 °C for 48 h. The resultant material was referred to as GNP-CTAC throughout the study. This surface modification process is simple, low-cost, and readily scalable, as it involves mild conditions, aqueous media, and no specialized equipment or intensive energy input. The procedure can be extended to larger batches by proportionally increasing reagent quantities and using conventional industrial mixing and drying systems. The pristine GNP was used as a reference for performance comparison. The characterization details are provided in Texts S1–S3.

### 2.3 Adsorption Experiments

The adsorption studies were conducted in a batch mode using 50 mL polypropylene centrifuge tubes from Corning Inc. (Corning, NY, USA). All tests were performed with three replicates. Each tube was supplemented with a mixture of eight perfluoroalkyl acids (PFAAs), namely C6–C10 perfluorocarboxylic acids (PFCAs), and C4, C6, C8 perfluorosulfonic acids (PFSAs); as well as two alternatives to PFOA and perfluorooctanesulfonic acid (PFOS), namely GenX and 6:2 fluorotelomer sulfonic acid (6:2 FTSA), respectively. The starting concentration of each PFAS in the adsorption performance evaluation experiments was 10 μg/L. Before adding an adsorbent at 100 mg/L, 500 μL of the PFAS solution was removed from each tube and used as an initial sample (0 min). Once the adsorbent was added, the tubes were put on a rotator shaker and set to revolve at 150 rpm. Samples were collected from each tube at 1, 2, 8, and 24 h. Following centrifugation, the supernatant was passed through 0.2 μm nylon syringe filters. The concentration of each PFAS was then analyzed using an Agilent Technologies 1290 Infinity II LC system coupled with a 6470 Triple Quad Mass Spectrometer (LC-MS/MS, Santa Clara, CA, USA). The effectiveness of the adsorbents in removing PFAS by adsorption was assessed using eq 1 in the following manner

1 $$\mathrm{removal}\;\mathrm{efficiency}\;\left(\%\right)=\frac{C_{i}-C_{t}}{C_{i}}\times 100$$

where *C*_*i*_ and *C_t_* are the PFAS concentration at initial and time (*t*), respectively.

Moreover, the details of adsorption kinetics, isotherm, and thermodynamics are described in Text S4.

### 2.4 Environmental Factors Studies

We evaluated the impact of three environmental factors on the adsorption of PFAS. The variables consisted of pH levels ranging from 2 to 12, different concentrations of natural organic matter (NOM) represented by humic acid from 2 to 100 mg/L, and different ionic strengths with NaCl concentrations ranging from 5 to 200 mM. Each PFAS was tested at 10 μg/L with an experimental duration of 4 h. Furthermore, the adsorption behavior of PFAS in river water was investigated using a similar approach, except that each PFAS was intentionally introduced at a concentration of 200 ng/L and exposed to the adsorbent for 4 h. The performance of GNP-CTAC on capturing the spiked PFAS was compared with that of a commercial adsorbent PAC from Calgon Carbon (Pittsburgh, PA, USA).

### 2.5 Interlaboratory Cross Validation Test

An interlaboratory cross-validation test was conducted at the New York State Center for Clean Water Technology at Stony Brook University, USA. The adsorption studies were conducted in a batch triplicate mode using a mixture of six PFAS, namely, PFOA, PFNA, PFBS, PFHxS, PFOS, and GenX, with a starting concentration of 10–15 μg/L and 100 mg/L of adsorbent dose. Samples were collected from each tube at 1, 12, and 48 h. GNP-CTAC, with two clay-based adsorbents modified with CTAC, modified clay (MC)^28^ and magnetic modified clay (MMC),^29^ was evaluated for comparison purposes.

### 2.6 Regeneration and Reuse of GNP-CTAC

The recyclability of GNP-CTAC was investigated through successive adsorption–desorption cycles. In the initial uptake experiment, 250 mg of pristine GNP-CTAC were agitated with 50 mL of a ten-component PFAS solution for 8 h. The adsorbent was intentionally dosed at 5 g/L in order to ensure enough and accurate mass measurement after repeated adsorption and desorption cycles. After centrifugation, the PFAS-loaded adsorbent was regenerated by three 30 min washes with 1% ammonium hydroxide/methanol and sonication, following the protocol outlined in EPA Method 1633.^22^ This regeneration step was repeated three times, and the refreshed adsorbent was subsequently exposed to fresh PFAS feed solutions for four additional adsorption cycles. For every cycle, the masses of PFAS introduced, captured, and recovered by the basic methanol rinse were determined as described in the Text S5. All methanolic extracts, aqueous rinses, and residual solutions were quantified by LC-MS/MS.

## 3 Results and Discussion

### 3.1 Structural Characterizations of GNP-CTAC

By adding CTAC surfactant, the ultrasonication method was able to stabilize and disperse GNP particles in water more effectively (Figure 1a), exhibiting a folded and crumpled structure with densely packed graphene layers (see the SEM image in Figure 1b). The EDS analysis (Figure 1c,d) revealed that carbon (C), oxygen (O), and chlorine (Cl) were the predominant elements, together constituting over 98% of the overall composition, aligning with previous studies on CTAC-intercalated graphene.^48^

**Figure 1:**
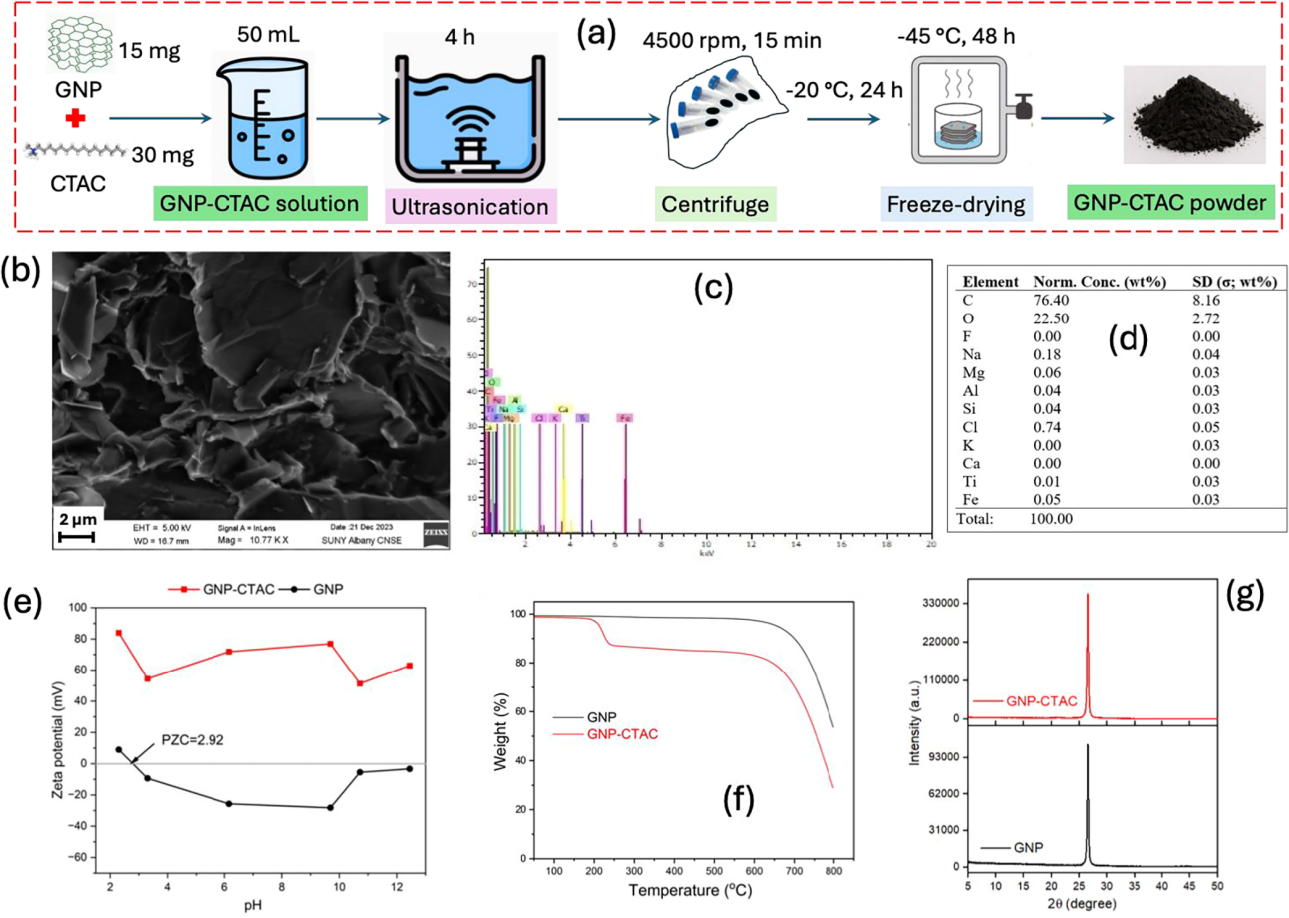
Schematic diagram (a), SEM (b), EDS spectra (c), EDS element composition (d), ζ potential (e), TGA (f), and XRD (g).

Figure 1e displays the ζ-potential of GNP and GNP-CTAC at various pH levels ranging from 2 to 13. The pristine GNP exhibited a positive surface charge in water only when the pH was below 3. Following the alteration of GNP by CTAC, the adsorbent consistently exhibited a positive point of zero charge over the entire pH range examined. This positive charge is advantageous for the adsorption of anionic PFAS.

The thermal stability of virgin GNP and GNP-CTAC was assessed in a nitrogen environment up to 800 °C (Figure 1f). Pristine GNP exhibited no substantial weight loss until over 650 °C, hence affirming its superior thermal resilience. The CTAC-intercalated composite demonstrated unique thermolysis characteristics. The first significant weight loss of GNP-CTAC occurred between 210 and 240 °C, attributed to the decomposition of oxygenated functional groups.^49^ The ongoing pyrolysis of CTAC resulted in a total weight loss of approximately 20% at 600 °C (Figure 1f). Given that environmental applications of these adsorbents are often conducted at ambient temperatures, GNP-CTAC exhibits adequate thermal stability for practical use.

Figure 1g displays the X-ray diffraction (XRD) patterns of GNP and GNP-CTAC. Pristine GNP had a pronounced peak at 2θ = 26.4°, indicative of the (002) basal reflection, with an interlayer spacing (*d*-spacing) of 3.370 Å, typical of the graphite layered structure.^50^ The lack of supplementary peaks validated the phase purity of the platelets. A comparable peak at 2θ = 26.4° was detected for GNP-CTAC, suggesting that CTAC intercalation did not modify the crystalline structure of GNP. The peak intensity for GNP-CTAC was much greater than that of pure GNP, suggesting improved stacking order or reduced defects of the graphene layers after CTAC modification, which may enhance the material’s efficacy in PFAS adsorption.^51^

The particle size decreased from 3.70 μm for GNP to 2.72 μm for GNP-CTAC after cationic alteration (Table S5). BET analysis revealed that the total surface area decreased from 20.89 m^2^/g for pristine GNP to 5.50 m^2^/g for GNP-CTAC, and the total pore volume reduced from 3 × 10^–2^ to 7.06 × 10^–3^ cm^3^/g. The maximum pore width slightly increased from 197.26 Å to 205.60 Å. These changes suggest that CTAC molecules partially filled the graphene pores, leading to reduced surface area and pore volume while slightly expanding the pore width. Similar trends have been reported in previous studies, where the addition of CTAC to GAC surfaces resulted in a reduction of the BET surface area and total pore volume, while concurrently enhancing bromate adsorption efficacy.^52^

### 3.2 Adsorption Performance

#### 3.2.1 Selective Adsorption

Figure 2a shows that the pristine GNP removed PFOS and PFDA to below detection after 1 h and PFNA in 2 h. On the other hand, short-chain PFAS showed no discernible decrease, while the removal percentages for PFHxS, PFOA, and 6:2 FTSA were only 50%. Despite the highly negatively charged surface of GNP, the presence of substantial adsorption suggests that PFAS were involved in nonelectrostatic interactions.^53^

**Figure 2:**
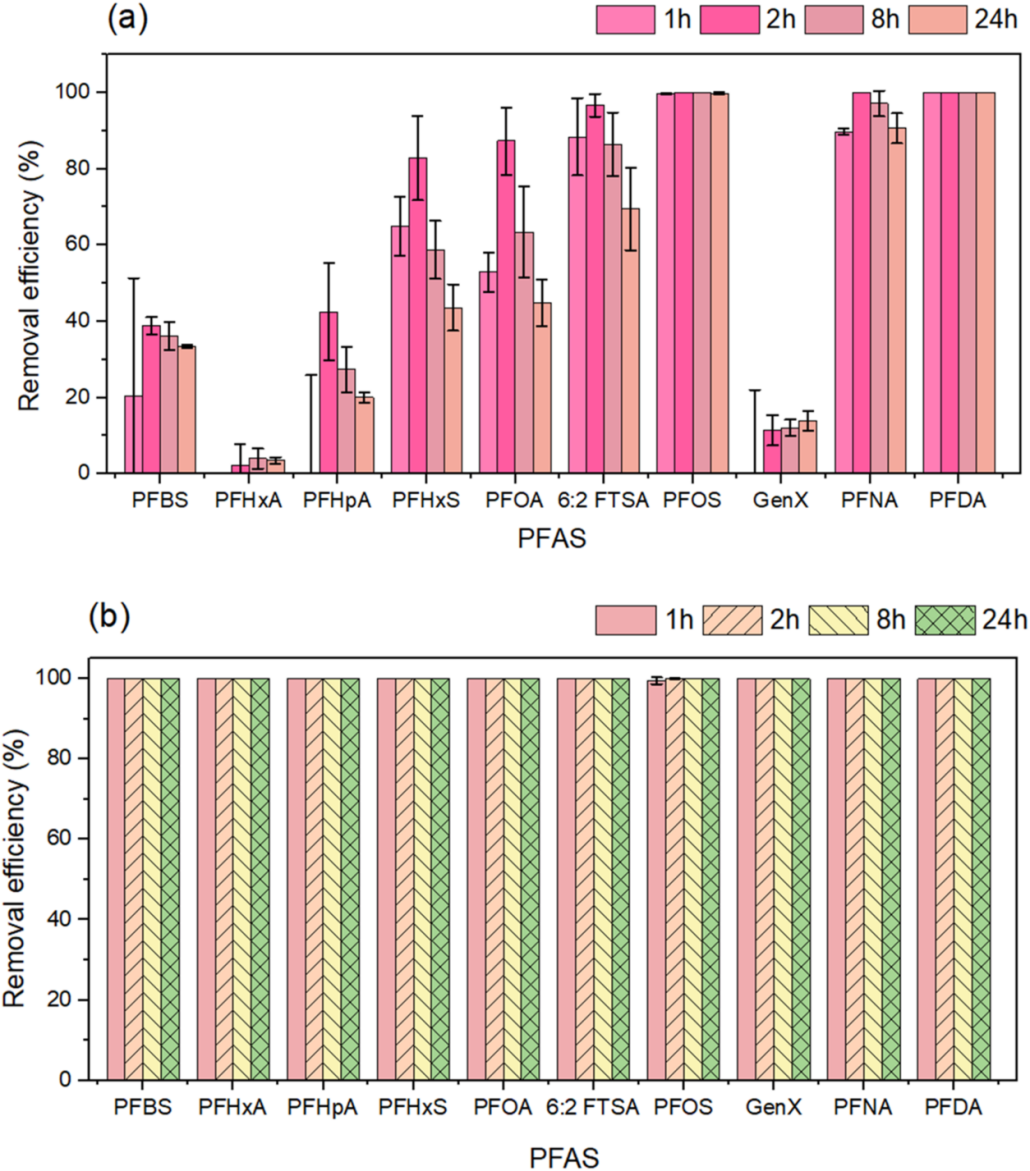
Adsorption of PFAS by (a) GNP and (b) GNP-CTAC. Experimental conditions were: initial PFAS concentration: 10 μg/L; adsorbent dose: 100 mg/L; sample collection points: 1, 2, 8, and 24 h. Error bars indicate the standard deviations of triplicate measurements.

Anionic PFAS can be quickly removed by cationic adsorbents due to their high electrostatic affinity, at least in principle.^54^ This was also demonstrated in a recent study by Maroli et al., in which the addition of CTAC significantly increased the removal of PFAS during conventional coagulation and flocculation processes.^55^ The addition of CTAC, a quaternary ammonium compound with a positive charge, to the GNP solution is in keeping with this. The resultant GNP-CTAC adsorbent was efficient in removing both short- and long-chain PFAS, with a removal efficiency of about 100% after just 1 h (Figure 2b). A cationic surfactant like CTAC may mediate the PFAS-molecular repulsion, allowing increased surface activity^56^ and micelle structure to develop at concentrations substantially below the critical micelle concentration. The aggregation effect of micelles then accelerated the PFAS adsorption capacity.^57,58^ Furthermore, GNP may represent a low-cost alternative to conventional adsorbents, highlighting its potential as a practical material for removing PFAS, particularly those with short chains.

#### 3.2.2 Kinetics, Isotherms, and Thermodynamic Studies

Figure 3a illustrates the batch adsorption kinetics of PFAS by GNP-CTAC. A rapid adsorption of total PFAS occurred during the first 10 s (Figure 3a, insert), achieving equilibrium under 1 min. The adsorption rate was significantly faster than the equilibrium timeframes previously reported for comparable batch systems (Table S6). The adsorption kinetics were assessed using the pseudo-first-order (PFO), pseudo-second-order (PSO), and intraparticle diffusion (IPD) models. Model fits were evaluated using their linearized representations, with the associated correlation coefficients (*R* ^2^) shown in Figures S1 and S2 for the 10 distinct PFAS. The PFO model generated an *R* ^2^ of 0.001, whereas the IPD model resulted in *R* ^2^ values ranging from 0.290 to 0.366. In contrast, the PSO model demonstrated an exceptional match, with an *R* ^2^ value of 1 for all PFAS analyzed (Figure S3). The results indicate that the adsorption kinetics of PFAS onto GNP-CTAC are optimally characterized by the PSO model, aligning with prior findings. Chen et al.^59^ found that the PSO model most accurately represented the adsorption of PFOA and PFOS onto carbonate-layered double hydroxides, whereas Omo-Okoro et al.^60^ also used the PSO model to evaluate PFAS adsorption. These data indicate that chemisorption is the primary process, characterized by electron sharing or exchange between PFAS and the active binding sites of GNP-CTAC.^61^

**Figure 3:**
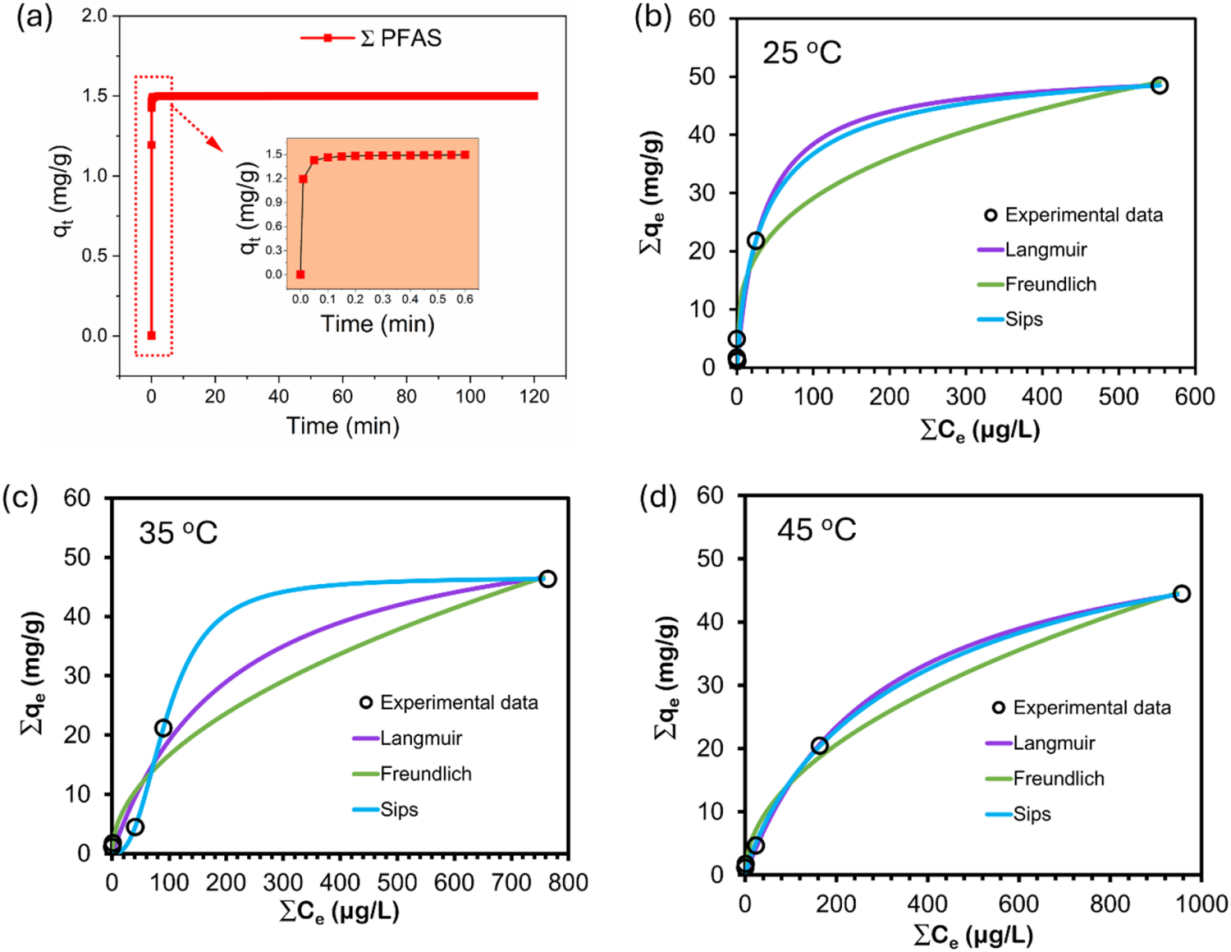
(a) Typical kinetic curve of total PFAS adsorption by GNP-CTAC at a starting concentration of 20 μg/L. Colored circles signify experimental data points, while solid lines illustrate model fits using the pseudo-second-order (PSO) model. Adsorption isotherms of total PFAS on GNP-CTAC were fitted using the Langmuir, Freundlich, and Sips models at (b) 25 °C, (c) 35 °C, and (d) 45 °C. All experimental results are presented as the average of three measurements.

Equilibrium isotherms are crucial for estimating the maximum adsorption capacity of adsorbents and for elucidating the interactions between PFAS and GNP-CTAC. This work used three prevalent isotherm models, i.e., Langmuir, Freundlich, and Sips, to analyze the adsorption characteristics of PFAS. The Langmuir isotherm characterizes monolayer adsorption on a uniform surface, as well as the equilibrium between adsorption and desorption, whereas the Freundlich isotherm addresses adsorption on nonuniform surfaces. The Sips model, a combination of Langmuir and Freundlich models, is especially appropriate for heterogeneous systems, as it encompasses both low- and high-concentration adsorption behaviors.

The experimental adsorption capacity (*q*_e_) and equilibrium concentration (*C*_e_) of total PFAS (ΣPFAS) were used for model fitting (Figure 3b). This method was essential because several individual PFAS were present at concentrations near or below detection limits after adsorption, preventing reliable isotherm determination for each compound. Theoretically and according to other researchers’ reports, there are competitions among short- and long-chain PFAS with different functional groups especially when the site for adsorption is limited.^62^ However, given the reality that different PFAS coexist in contaminated environments, studying adsorption of PFAS in singular, binary or trinary systems does not reveal what would happen in the field. Thus, in reported studies^63–65^ and in this study, all adsorption experiments were conducted in a multisolute system in order to better simulate realistic environmental waters where PFAS coexist. Figure S4 illustrates the correlation between the initial concentration (*C*_0_) and the quantity adsorbed, while Figures 3b–d and Tables S7–S10 present the model fits. The Langmuir model accurately represented adsorption behavior at high concentrations, but the Freundlich model demonstrated a more effective match at diminished concentrations. Throughout the entire concentration spectrum, the Sips isotherm provided the most precise approximation, yielding a total adsorption capacity of 54.08 mg/g for the PFAS mixture and surpassing the constraints of the Freundlich model at high concentrations. Since all adsorption experiments were conducted in a multisolute system, the obtained capacity inherently reflects the competitive adsorption behavior among the ten investigated PFAS. Such competition likely arises from differences in chain length, hydrophobicity, and functional group type. At low adsorbate concentrations, the Sips isotherm exhibited characteristics similar to those of the Freundlich model; however, at elevated levels, it mirrored the Langmuir model, thereby integrating the advantages of both. The elevated R^2^ values for the Sips model validated its enhanced predictive capability, aligning with previous research on PFAS adsorption.^66,67^

Temperature further affected adsorption efficacy. Figure 3b,3c illustrate that the adsorption capability of GNP-CTAC decreases with increasing temperature, indicating that the process is exothermic.^68^ The thermodynamic parameters (Δ*G*, Δ*H*, and Δ*S*) derived from the isotherm data (Table S7) corroborated this finding. The negative Δ*G* values affirmed the spontaneity of PFAS adsorption, but the positive Δ*H* and Δ*S* values indicated heightened unpredictability at the solid–solution interface.^69^

Notably, in contrast to previous research that has mostly focused on a restricted range of PFAS at mg/L concentrations, GNP-CTAC demonstrated a robust ability to adsorb combinations of both short- and long-chain PFAS at ecologically relevant μg/L values. Moreover, no desorption of PFAS was observed throughout the 24-h testing period, underscoring the durability of the adsorption process. The data indicates that GNP-CTAC is an effective material for removing complex PFAS combinations under actual environmental conditions.

#### 3.2.3 Effect of pH

The pH of a solution is crucial in influencing the surface charge properties of an adsorbent and the speciation or behavior of adsorbates.^70^ Regarding PFAS removal with GNP-CTAC, Figure 4a illustrates that adsorption was highly efficient across an extensive pH range (2–12). The reliable performance is attributed to the persistent positive surface charge of GNP-CTAC across various pH levels, which facilitates electrostatic adhesion to negatively charged PFAS. Prior research has also shown that adsorbents with a greater positive surface charge exhibit an increased affinity for PFAS.^71^

**Figure 4:**
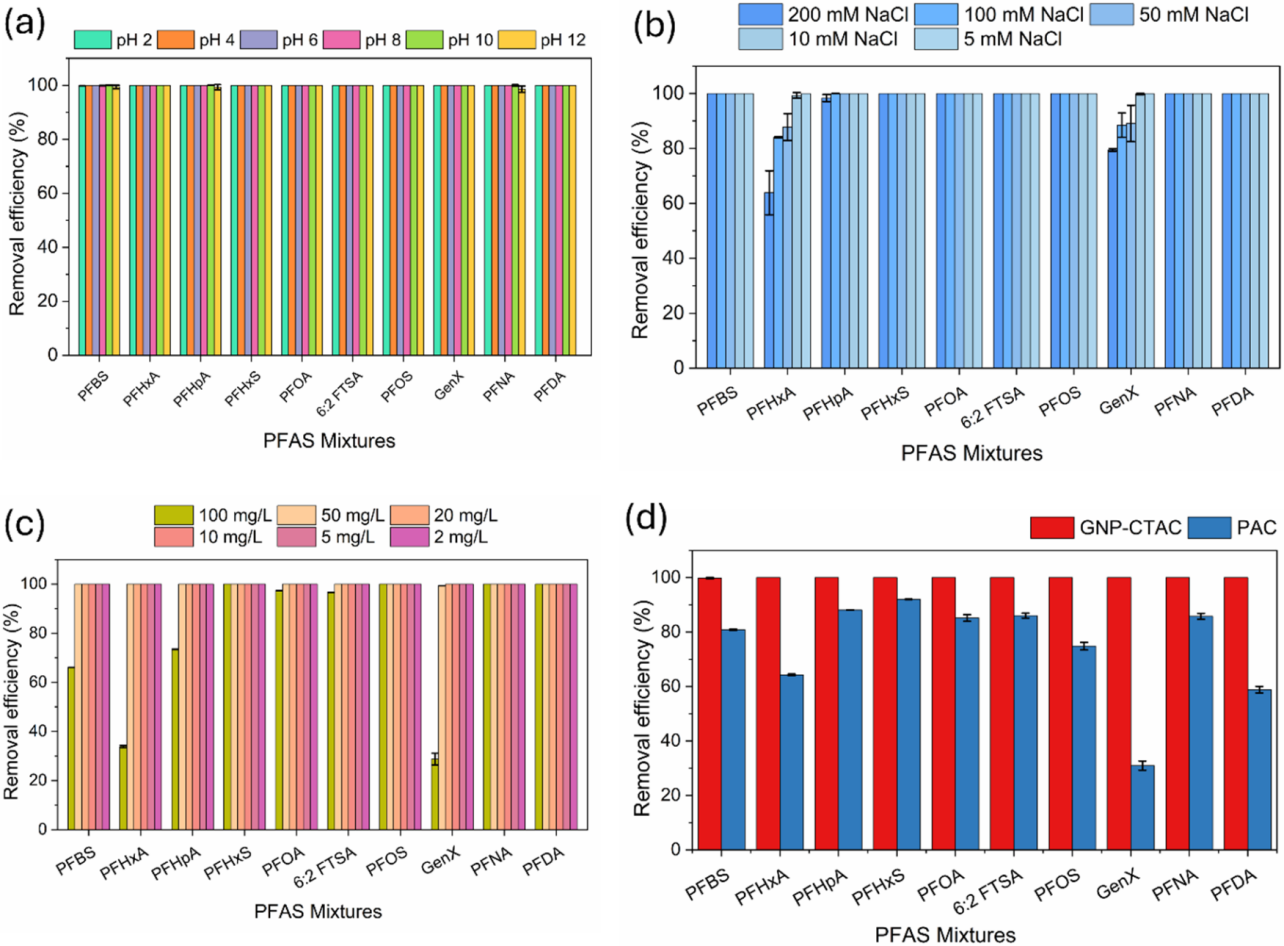
Effects of (a) pH, (b) NaCl, and (c) NOM on PFAS removal (10 μg/L), and (d) adsorption by GNP-CTAC and PAC in Hudson River water (200 ng/L). Adsorbent dose: 100 mg/L; contact time: 4 h. Error bars show triplicate standard deviations.

The consistent removal efficacy of GNP-CTAC across acidic, neutral, and alkaline environments indicates that hydrophobic interactions, in conjunction with electrostatic forces, significantly contribute to PFAS adsorption. The significant adsorption observed at ecologically pertinent pH ranges (5–8.5), characteristic of most natural waters,^72^ further emphasizes the capability of GNP-CTAC as an effective adsorbent for PFAS cleanup in various aquatic settings.

#### 3.2.4 Effect of Ionic Strength

The effect of ionic strength on PFAS adsorption by GNP-CTAC was examined by varying NaCl concentrations from 5 to 200 mM (Figure 4b). Given the fact that many cations and anions are potentially present in environmental water and the impossibility of evaluating each one on their effect on PFAS adsorption, NaCl has been generally used to represent ionic strength contributed by different ions.^24,29,73^ For short-chain PFAS such as perfluorohexanoic acid (PFHxA) and GenX, adsorption efficiency declined as ionic strength increased from 50 to 200 mM. This trend is consistent with previous findings, which suggest that cations can associate with the negatively charged functional groups of PFAS, forming weak neutral complexes that reduce electrostatic attraction to the adsorbent surface.^74,75^

In contrast, the adsorption of long-chain PFAS remained unaffected, with complete removal observed even at 200 mM NaCl. This outcome aligns with the pH-dependent results, providing further evidence that hydrophobic interactions dominate the adsorption of long-chain PFAS on GNP-CTAC, whereas short-chain PFAS rely more heavily on electrostatic interactions. Importantly, the effective removal of PFAS across a wide ionic strength range (5–200 mM) highlights the robustness of GNP-CTAC. Given that ionic strengths typically range from 1–5 mM in surface waters, 1–20 mM in groundwater, and up to ∼700 mM in seawater,^76^ these findings suggest that GNP-CTAC is a promising adsorbent for PFAS remediation in diverse aquatic environments, including high-salinity systems. It is noteworthy that NaCl was used as a representative electrolyte due to the prevalence of Na^+^ and Cl^–^ in natural waters. However, divalent ions (e.g., Ca^2+^, Mg^2+^) may induce stronger electrostatic screening or competition due to their higher charge density and potential cation bridging effects,^77,78^ probably altering PFAS adsorption behavior. Future investigations involving multiple electrolytes are warranted to further elucidate these effects.

#### 3.2.5 Effect of NOM

Surface waters and wastewaters typically contain significant amounts of natural organic matter (NOM), which can influence the adsorption of PFAS. As shown in Figure 4c, GNP-CTAC maintained strong adsorption performance across humic acid concentrations ranging from 2 to 100 mg/L for most PFAS. However, at a humic acid concentration of 100 mg/L, a reduction in adsorption was observed for PFBS, PFHxA, PFHpA, and GenX. This decrease suggests competitive interactions between NOM and these short-chain PFAS for active adsorption sites on the GNP-CTAC surface. Similar inhibitory effects of NOM on PFAS adsorption have been reported previously, for example, with boehmite, where organic matter substantially reduced the adsorption efficiency.^79^

The inhibitory role of NOM can be attributed to two primary factors: (i) competition for adsorption sites due to its large molecular size and surface activity, and (ii) electrostatic repulsion between the negatively charged NOM and PFAS anions, which may hinder PFAS mobility toward the adsorbent surface. Despite this, GNP-CTAC still achieved nearly complete removal of all PFAS even at the highest humic acid concentration tested (100 mg/L). This result highlights the effectiveness of cationic modification in enhancing the adsorption capacity of GNPs, enabling them to outcompete NOM for binding sites. Considering that typical NOM levels in surface waters are within the range of 2–10 mg/L,^80,81^ these findings suggest that GNP-CTAC can reliably remove PFAS under environmentally relevant conditions, even in NOM-rich aquatic systems.

#### 3.2.6 Actual River Water

To assess the practical application of GNP-CTAC, adsorption studies were performed using Hudson River water with background PFAS concentrations ranging from 5 to 25 ng/L (Table S4).^24^ The adsorption experiments were conducted at a spiked concentration of 200 ng/L for each PFAS. It needs to be noted that this study did not seek to remove PFAS in the samples collected from the Hudson River. The reason for using Hudson River samples was to evaluate the adsorptive performance of the GNP-CTAC material toward removing PFAS in the real water matrix where other cations, anions and organic matters are present. The spiked concentration of 200 ng/L was to simulate contaminated water in the real world where PFAS concentrations are in the range of hundreds of ng/L to μg/L levels. As shown in Figure 4d, complete elimination was accomplished within 4 h for each PFAS. Conversely, a commercial PAC (REMPAC from Calgon Carbon) exhibited much worse adsorption efficacy under the same circumstances. This indicates that the presence of coexisting species in the river water significantly diminished the efficacy of PAC, but GNP-CTAC maintained its high adsorption ability.

The analysis of Hudson River water indicated a bromide content of 5.64 × 10^–3^ mg/L, a phosphate concentration of 7.60 × 10^–3^ mg/L, and total nitrogen (TN) values of 1.00 mg/L (Table S4), which exceed the concentrations often seen in precipitation or runoff.^82,83^ The total organic carbon (TOC) content of 5.578 mg/L aligns with worldwide averages for surface waters.^84^ Both anions and NOM are recognized to compete with PFAS for adsorption sites, suggesting that these background elements likely decrease the efficacy of PAC.^61,85^ In contrast to previous research indicating reduced PFAS removal by conventional adsorbents in natural waters,^86,87^ GNP-CTAC exhibited superior adsorption effectiveness under ecologically relevant conditions. The findings indicate that GNP-CTAC is resilient to competing solutes and outperforms PAC, highlighting its significant potential for PFAS cleanup in intricate surface water matrices.

### 3.3 Interlaboratory Cross Validation

The results of the interlaboratory comparison of the three CTAC-modified adsorbents, i.e., MC, MMC, and GNP-CTAC, are presented in Figure 5a–c. The data revealed that MC demonstrated satisfactory adsorption performance, achieving nearly 100% removal efficiency of the PFAS mixture, except for GenX, which was removed at an efficiency of approximately 97% at 48 h. MMC had higher adsorption performance than MC, achieving nearly 100% removal efficiency across all PFAS mixtures. All these results confirmed what we reported previously regarding MC and MMC.^28,29^ Similar to MC and MMC, the GNP-CTAC achieved an almost 100% removal efficiency for PFBS, PFHxS, PFOA, PFOS, and PFNA. Its removal of GenX, however, was only 87%, which was lower than that of MC and MMC. Interestingly, the GNP-CTAC had nearly 100% capture of GenX in spiked Hudson River water samples at around 200 ng/L (Figure 4d). Thus, the GNP-CTAC appears promising for removing PFAS in real environmental water samples and warrants further evaluation.

**Figure 5:**
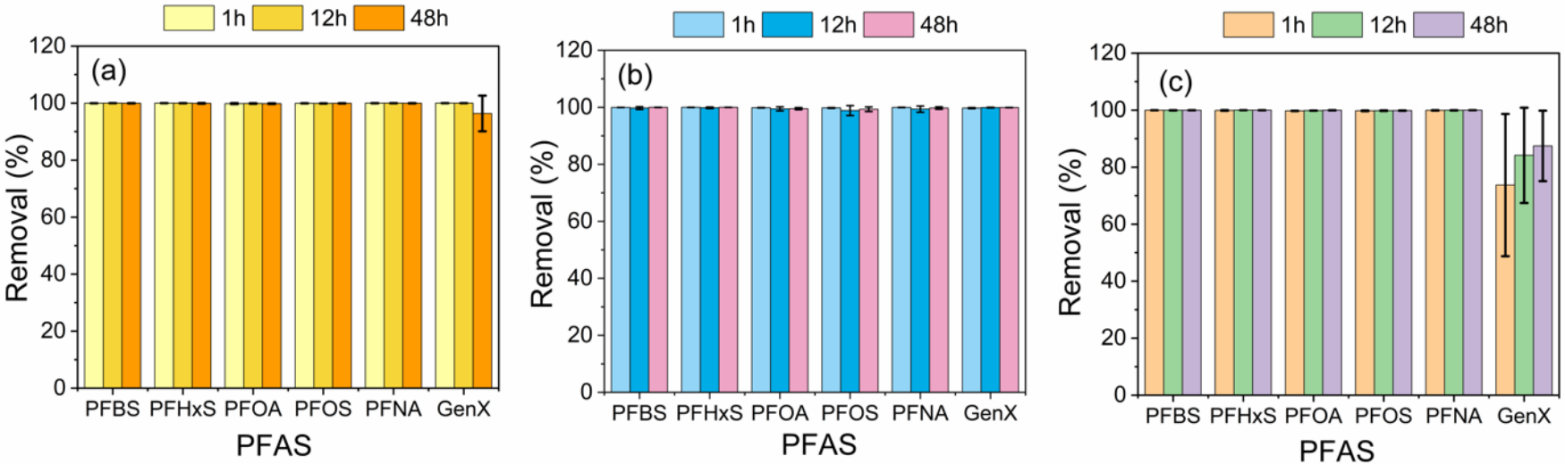
Interlaboratory cross-validation test for (a) MC, (b) MMC, and (c) GNP-CTAC. Experimental conditions were: initial PFAS concentration: 10–15 μg/L in pure water; adsorbent dose: 100 mg/L; sample collection points: 1, 12, and 48 h. Error bars indicate the standard deviations of triplicate measurements.

### 3.4 Regeneration and Reuse

Commercial viability of PFAS adsorbents relies heavily on their regenerability and reusability. In this study, multiple regeneration and reuse cycles were used to investigate the adsorption performance of GNP-CTAC and determine its reusability. The effectiveness of GNP-CTAC regeneration and the adsorption behavior across four cycles for a combination of 10 PFAS are shown in Figure 6. The capturing efficiency for the short-chain PFAS, namely PFHxA, PFBS, and GenX, dropped from nearly 100% to 46.79 ± 0.63%, 52.61 ± 1.11%, and 59.07 ± 0.40%, respectively, after four cycles’ regeneration using 1% basic methanol. This decrease may be due to the leakage or release of CTAC from the adsorbent, which could lead to decreased electrostatic interactions between the adsorbent and the short chain PFAS. To enable strong binding between relatively hydrophilic PFAS and the GNP-CTAC over multiple cycles, one remedy approach is to soak the adsorbent in a CTAC solution after regeneration. This will be explored in future studies.

**Figure 6:**
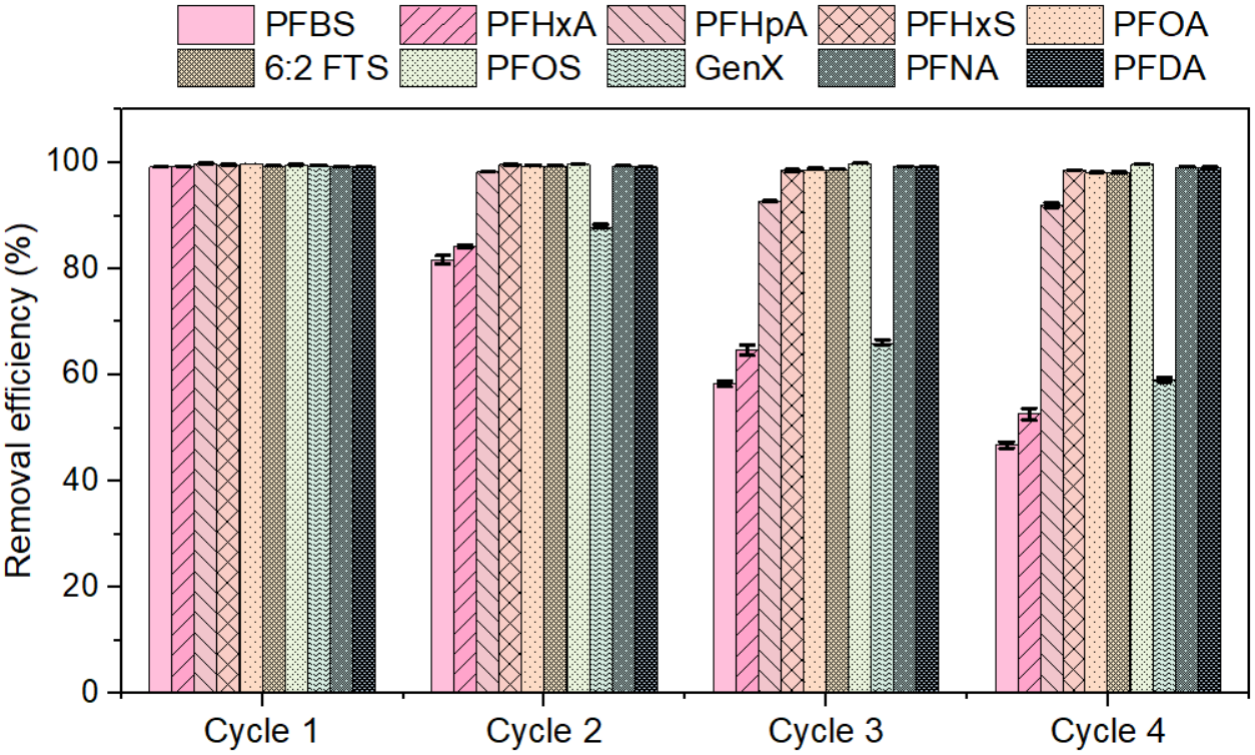
Removal % of PFAS mixtures after four cycles of regeneration and reuse. Initial PFAS concentration: 10 μg/L, GNP-CTAC’s dose: 5 g/L, time: 8 h; solution volume: 50 mL, and rpm: 150 at 25 °C. Error bars represent the standard deviations of triplicate measurements.

Past efforts to recycle carbon-based adsorbents mostly used thermal and nonthermal methods, including chemical regeneration, microwave irradiation, ultrasonic treatment, and high temperatures between 800–1000 °C,^88^ and the reusability was also not very high (only 65% efficiency was observed after 5 cycles).^89^ It is believed that structural changes, such as irreversible adsorption or pore blockage, are to blame for the declining adsorption capacity with increasing cycles.^90^ In this study, given the decreased removal of short-chain PFAS with increasing regeneration and reuse cycles, better regeneration methods need to be identified to use GNP-CTAC in multiple cycles while keeping its adsorption performance stable. Moreover, the secondary waste solution produced during regeneration requires proper treatment before disposal. Advanced oxidative and reductive destruction technologies, such as photodegradation,^91–93^ electrochemical oxidation,^94^ or plasma-based treatment,^95^ could be applied to mineralize PFAS in the waste stream.

### 3.5 Adsorption Mechanism

#### 3.5.1 FTIR

The functional groups of GNP, GNP-CTAC, and PFAS-loaded GNP-CTAC were analyzed using FTIR spectroscopy (Figure 7a). The FTIR spectra of virgin GNP displayed notable bands at 1100 cm^–1^ (C=O stretching), 1490 cm^–1^ (C–O group), 2990 and 2850 cm^–1^ (−CH_2_ groups), and 3450 cm^–1^ (−OH group), therefore affirming the existence of oxygen-containing surface functionalities.^96–98^ The GNP-CTAC composite exhibited all the distinctive peaks of GNP, in addition to further characteristics ascribed to CTAC. Notably, robust absorption bands at 1240 and 1280 cm^–1^ were attributed to aromatic C–C and C–N stretching vibrations, while the significant band at 1750 cm^–1^ was ascribed to C=O groups affected by CTAC intercalation.^99^ Following PFAS adsorption, the total FTIR signal strength of GNP-CTAC significantly decreased. The extensive area between 1500–1000 cm^–1^ reduced to a single band, aligning with documented interactions between the fluorinated and sulfonate groups of PFAS and the GNP-CTAC surface.^100^ Furthermore, the hydrophobic −CH_2_– bands of GNP-CTAC were markedly diminished after PFAS adsorption, suggesting that the hydrophobic regions of PFAS interacted with the alkyl chains of CTAC. The concurrent decrease in −CH_2_– vibrational intensity and the strong affinity of GNP-CTAC toward both long- and short-chain PFAS together indirectly indicate the involvement of hydrophobic interactions. However, direct measurements, such as contact angle or surface energy analyses, are warranted to further elucidate the hydrophobic contribution in future studies. Importantly, the persistence of the characteristic CTAC peaks (C–N stretching at 1240–1280 cm^–1^ and C=O at 1750 cm^–1^) after PFAS adsorption confirms that CTAC remained stably attached to the GNP surface, with no evidence of surfactant leaching during the adsorption process.

**Figure 7:**
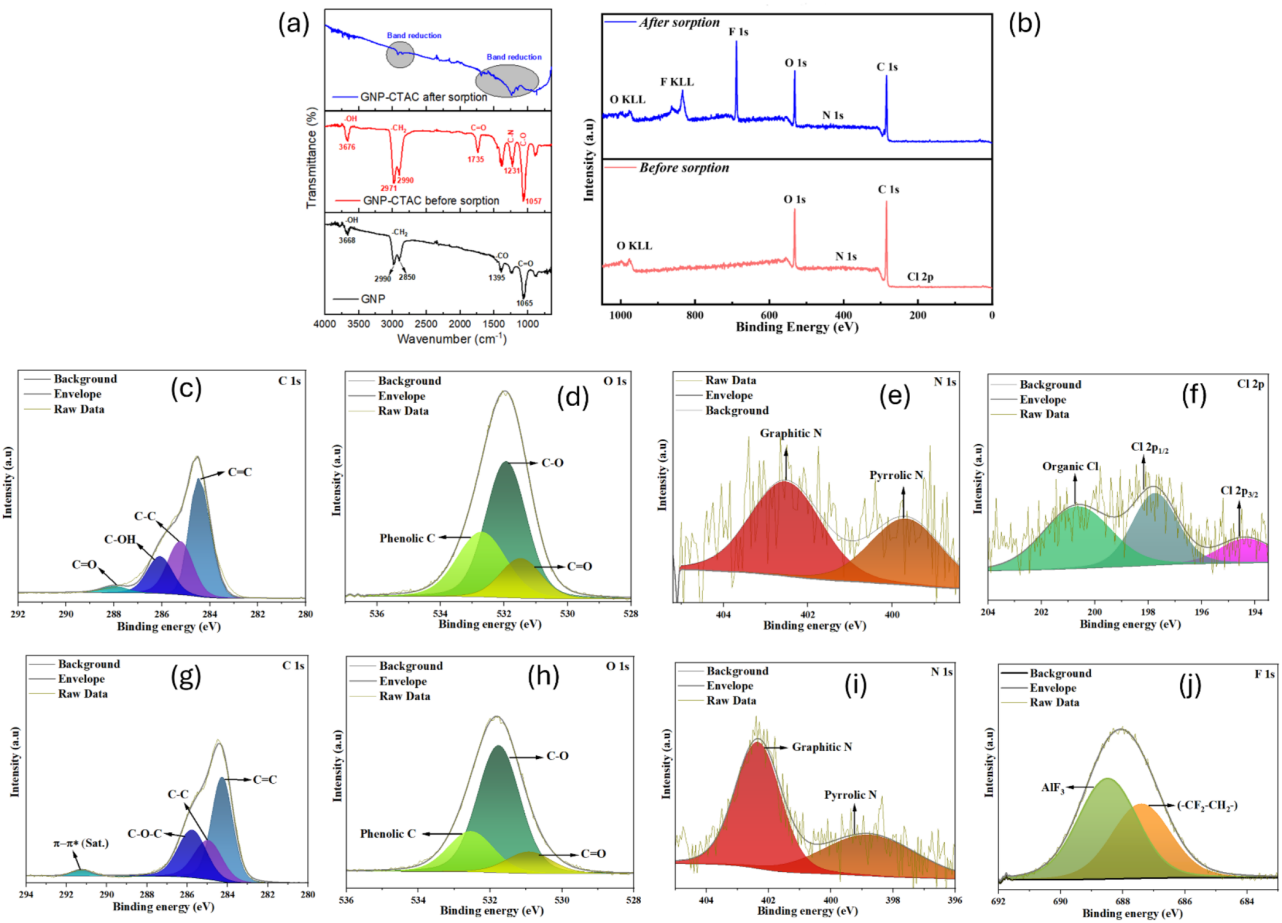
(a) FTIR spectra of the GNP and GNP-CTAC (before and after adsorption), (b) XPS survey, (c–f) C 1s, O 1s, N 1s, and Cl 2p spectra of GNP-CTAC before adsorption and (g–j) after adsorption.

#### 3.5.2 XPS

Figure 7b displays the X-ray photoelectron spectroscopy (XPS) survey spectra of GNP-CTAC before and after PFAS adsorption. The investigation offered insights into the surface chemistry and adsorption mechanisms of PFAS on the composite. Table S11 indicates that the predominant constituents on the GNP-CTAC surface were carbon (79.28%), oxygen (19.57%), and nitrogen (0.69%). The detection of a Cl 2p signal with a relative abundance of 0.46% corroborated the efficient integration of quaternary ammonium groups into the graphene surface. Following PFAS adsorption, a notable F 1s signal was seen with a relative abundance of 6.78%, providing direct evidence of the great affinity of GNP-CTAC for PFAS binding.

High-resolution XPS spectra of C 1s, O 1s, N 1s, Cl 2p, and F 1s for GNP-CTAC before and after PFAS adsorption are shown in Figure 7c–f and g–j, respectively. In the pristine GNP-CTAC, the C 1s spectrum exhibited peaks at 284.6, 285.3, 286.2, and 288.2 eV, corresponding to C=C, C–C, C–OH, and C=O bonds, respectively. Following adsorption (Figure 7g), the peak at 286.2 eV was reassigned to C–O–C, a feature characteristic of graphene frameworks that overlaps with C–OH signals.^101^ A weak signal at 291.5 eV, associated with the π–π* shakeup transition, further indicated aggregation of CTAC molecules on the graphene surface through π–π interactions.^102^ Clear variations were also observed in the O 1s spectra (Figure 7d,7h). The binding energies at 531.8 and 532.7 eV were attributed to C=O and C–O groups located at the graphene sheet edges.^103^

The N 1s spectrum provided further evidence of surface modification and PFAS adsorption. A peak at 400.2 eV confirmed the presence of C–N bonding, which is typically associated with the availability of lone pairs.^104^ In GNP-CTAC, an additional peak at 402.3 eV was observed, corresponding to quaternary ammonium groups introduced during CTAC modification (Figure 7e). After PFAS adsorption, the C–N peak shifted from 400.2 to 399.4 eV (Figure 7i), indicating a change in the electronic environment of nitrogen. This shift suggests interactions between the amine functionalities of GNP-CTAC and PFAS, consistent with adsorption via electrostatic attraction and chemical bonding.

The Cl 2p spectra of GNP-CTAC (Figure 7f) exhibited two peaks at 197.7 and 195.6 eV, corresponding to the Cl 2p_1/2_ and Cl 2p_3/2_ transitions, respectively. The 2.1 eV interval between the peaks is indicative of chloride ions bound to quaternary ammonium groups.^105^ The atomic percentage of chlorine decreased from 0.39 to 0.07% after PFAS adsorption, indicating partial ion exchange throughout the process. The F 1s spectra (Figure 7j) displayed a pronounced peak at 688.7 eV, unequivocally validating the successful adsorption of PFAS onto GNP-CTAC. This discovery indicates that ion exchange had a role in the adsorption process. Nonetheless, as shown by the NaCl competition assays (Figure 4b), the effectiveness of PFAS removal was not significantly influenced by the presence of chloride ions, underscoring that other mechanisms—such as hydrophobic interactions and electrostatic attraction—were also relevant (Figure 8). The findings together demonstrate that ultrasonication decreased particle size, but CTAC functionalization conferred a positive surface charge to GNP-CTAC. The multilayered graphene structure significantly promoted CTAC intercalation and enhanced the efficacy of PFAS adsorption.^41,106^

**Figure 8:**
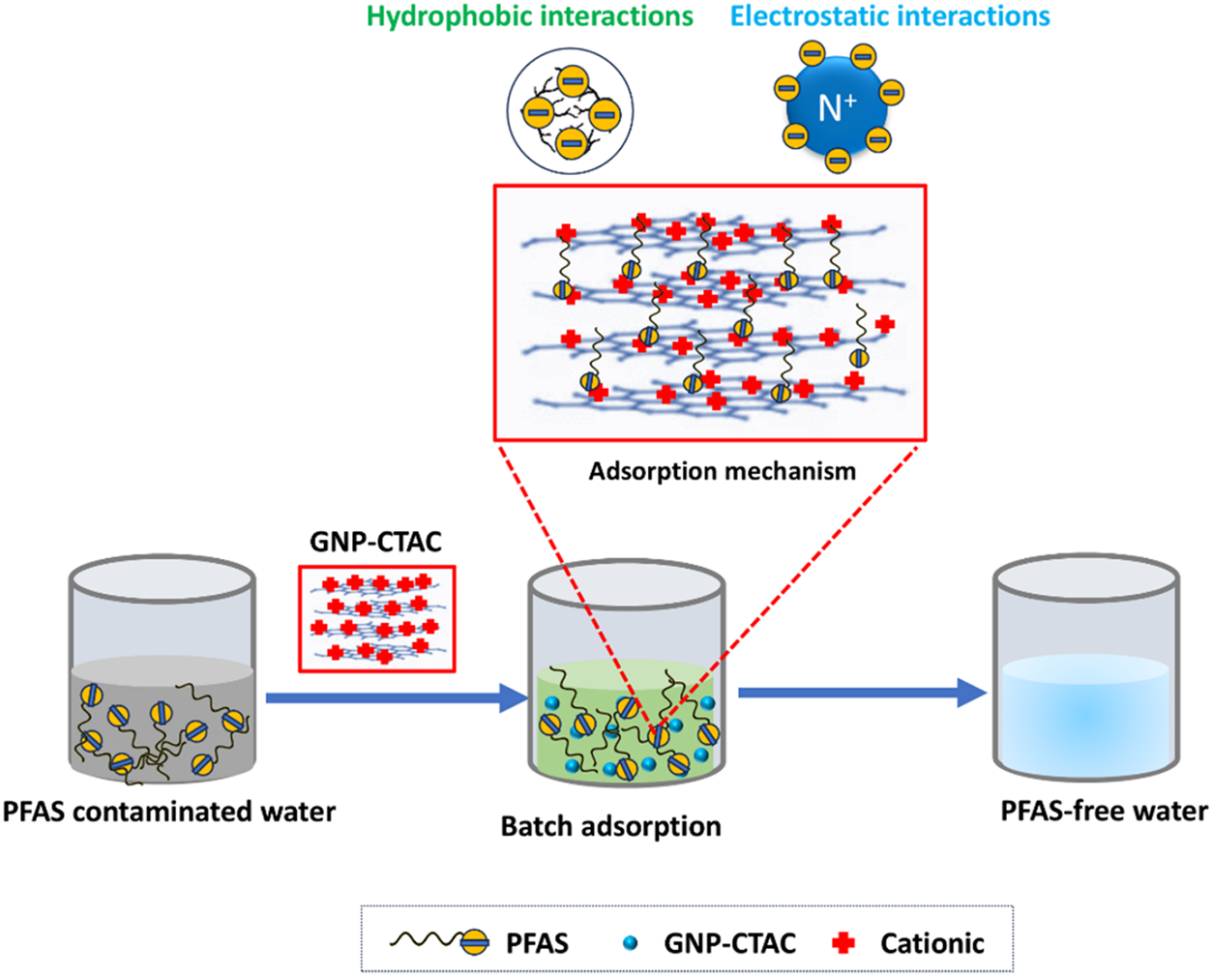
Schematic illustration of PFAS adsorption mechanism on GNP-CTAC.

To further confirm the stability of the adsorbent during four adsorption-regeneration-reuse cycles, sequential FTIR and SEM analyses were performed. In FTIR (Figure S5), a band with similar characteristics was detected after each regeneration cycle, and band reduction was noticed after each adsorption cycle. These phenomena can be correlated with the previous analysis in Figure 7a. Furthermore, the SEM analysis confirmed that the adsorbent’s morphology remained stable (without CTAC leaching) during adsorption, regeneration, and reuse cycles (Figure S6).

## 4 Conclusions

This study reports the synthesis of a new graphene nanoplatelets-based material, GNP-CTAC, and demonstrates its high adsorption capacity for removing PFAS from water. Extensive characterizations revealed that the CTAC was effectively loaded onto the GNP surface without significant alterations to its structure. The inclusion of CTAC resulted in a significantly positive surface charge for the GNP-CTAC adsorbent, which played a crucial role in enhancing the adsorption effectiveness. The findings demonstrated that GNP-CTAC achieved near-complete capture of all target PFAS in pure water within an hour and in river water samples within 4 h. The efficacy of PFAS removal was little impacted by variations in pH, NOM concentration, and ionic strength. The adsorption of PFAS by GNP-CTAC could be properly represented using a Sips isotherm model, with a calculated adsorption capacity of 54.08 mg/g. The adsorption processes of GNP-CTAC were postulated based on its physicochemical features and the investigation of the environmental effect on PFAS adsorption. These mechanisms include electrostatic and hydrophobic interactions. Furthermore, the GNP-CTAC demonstrated remarkable reusability throughout four adsorption and desorption cycles of PFAS in water. This work introduces a novel, low-cost, efficient nanoscale-based graphene adsorbent that contributes to the quest for suitable materials to remove PFAS from different water conditions.

## Supplementary Material



## Notes

Characterization (Text S1); Analytical methods (Text S2); detailed procedures for PFAS analysis (Text S3); adsorption kinetics, isotherms, and thermodynamic studies (Text S4); mass calculation of PFAS added, removed by adsorption, and recovered by regeneration (Text S5); chemicals and reagents used in this study (Table S1); the physicochemical properties of PFAS used in this study (Table S2); dynamic multiple reaction monitoring transitions for the studied PFAS (Table S3); characteristics of the investigated Hudson River water (Table S4); particle size, BET total surface area, and pore size distribution analysis (Table S5); adsorption performance comparison of the prepared GNP-CTAC with other similar adsorbents reported in the literature (Table S6); fitted isotherm modeling parameters for PFAS adsorption on GNP-CTAC (Table S7); experimental and isotherm modeled values of total adsorbed PFAS at equilibrium (*q*_e_) and concentrations in aqueous phase at equilibrium (*C*_e_) in the adsorption process by GNP-CTAC at 25 °C (Table S8); experimental and isotherm modeled values of total adsorbed PFAS at equilibrium (*q*_e_) and concentrations in aqueous phase at equilibrium (*C*_e_) in the adsorption process by GNP-CTAC at 35 °C (Table S9); experimental and isotherm modeled values of total adsorbed PFAS at equilibrium (*q*_e_) and concentrations in aqueous phase at equilibrium (*C*_e_) in the adsorption process by GNP-CTAC at 45 °C (Table S10); summary of binding energy and atomic concentration of GNP-CTAC (Table S11); fitting of adsorption data of PFAS at the initial concentrations of 10 μg/L by the linear form of pseudo-first-order model (Figure S1); fitting of adsorption data of PFAS at the initial concentrations of 10 μg/L by the linear form of intraparticle diffusion model (Figure S2); fitting of adsorption data of PFAS at the initial concentrations of 10 μg/L by the linear form of pseudo-second-order model (Figure S3); the relationship between initial concentrations (*C*_0_) and mass of PFAS adsorbed by the GNP-CTAC (Figure S4); FTIR spectra after four cycles of regeneration and reuse (Figure S5); SEM analysis during four adsorption-regeneration cycles (Figure S6) (PDF)
