# Supplementary material for: Regenerable Graphene Nanoplatelet Adsorbents for Rapid and Trace-Level PFAS Removal from Water
Source: ACS Omega. 2026 Jan 5;11(2):2902–16. doi: 10.1021/acsomega.5c08997 (PMC12824806; doi:10.1021/acsomega.5c08997)
Supplement: Supplementary file 1 [file ao5c08997_si_001.pdf]

Supporting Information for

Regenerable graphene nanoplatelet adsorbents for rapid and trace-level PFAS removal from water

Md. Nahid Pervez [a], Tao Jiang [a, *], Aswin Kumar Ilango [a], Yamini Kumaran [b], Yi Zhang [c], Weilan Zhang [a], Haralabos Efstathiadis [b], Jeremy I. Feldblyum [d], Mehmet V. Yigit [d], Arjun K. Venkatesan [e], Yanna Liang [a, *]

[a] *Department of Environmental and Sustainable Engineering, University at Albany, State University of New York, Albany, NY 12222, USA*

[b] *Department of Nanoscale Science and Engineering, University at Albany, State University of New York, Albany, NY 12222, USA*

[c] *New York State Center for Clean Water Technology, Stony Brook University, Stony Brook, NY 11794, USA*

[d] *Department of Chemistry, University at Albany, State University of New York, Albany, NY 12222, USA*

[e] *Department of Civil and Environmental Engineering, New Jersey Institute of Technology, Newark, NJ 07102, USA*

*** Corresponding authors:**

tjiang2@albany.edu (T. Jiang)

yliang3@albany.edu (Y. Liang)

Text S1: Characterization

A scanning electron microscope integrated with energy dispersive X-ray spectroscopy (SEM-EDS, Zeiss LEO 1550, Oberkochen, Germany; Bruker Quantax XFlash 6, Billerica, MA, USA) was used for the morphological and compositional examinations of the materials. Functional group analysis in the adsorbent samples before and after PFAS adsorption was carried out using Fourier transform infrared spectroscopy (FTIR; PerkinElmer Spectrum 100, Waltham, MA, USA). With a resolution of 1 cm^{-1}, the spectral data were acquired within the 4,000-650 cm^{-1} spectral band. Thermogravimetric analysis (TGA) was conducted using a TGA-5500 instrument (New Castle, DE, USA) to assess the adsorbents' thermal behavior. Nitrogen gas adsorption isotherms were obtained at a temperature of 77 K using a 3Flex gas adsorption analyzer (Micromeritics, Norcross, GA, USA). Before the N_2 gas adsorption examination, the adsorbents underwent activation under decreased pressure and at a temperature of 50 °C (about 0.1 mbar) for 24 h. The Brunauer Emmet Teller (BET) technique was used to estimate the surface areas, while the pore size distributions were derived using density functional theory. The XRD was fitted with a graphite monochromator and a D/teX Ultra one-dimensional silicon strip detector. The investigated crystalline samples were pulverized and positioned in zero-background holders, which were subjected to scanning with a 0.01° increment. The Malvern Zetasizer Nano-ZS analyzer (Malvern Panalytical Ltd, Malvern, UK) was used to measure the particle size distribution and ζ potential at a neutral pH and room temperature. The chemical status of the surface elements was analyzed using X-ray photoelectron spectroscopy (XPS) using the PHI Quantera II instrument. The Shirley Background method was used for the purpose of peak fitting, with the C-C peak at

284.7 eV serving as the reference peak. The detailed chemical and PFAS analysis information is presented in **Text S2, Text S3, and Table S3.**

Text S2: Analytical methods

PFAS in river water samples were measured in accordance with EPA Method 537.1. Each 400 mL sample was augmented with 30 µL of ^{13}C-perfluorohexanoic acid (PFHxA, 1 mg/L; 30 ng total) as a surrogate. The solution was then processed with HyperSep C18 solid-phase extraction (SPE) cartridges that had been preconditioned with methanol and deionized water. Following extraction, the eluate was supplemented with ^{13}C-PFOS and ^{13}C-PFOA internal standards. PFAS chemicals were quantified using an Agilent LC-MS/MS system. In adsorption trials, the supernatant was examined similarly, except for the SPE stage. Methodological specifics and validation are detailed in other papers[1-5].

The anionic content of the river water was analyzed using ion chromatography with a 930 Compact IC Flex system (Metrohm, Switzerland) equipped with a conductivity detector. A Metrosep SUPP 5 column was used with a carbonate/bicarbonate eluent (1.8 mM Na_2CO_3 and 1.7 mM $NaHCO_3$, 1:1 v/v) at a flow rate of 0.7 mL/min. A 0.05 M H_2SO_4 solution was used as a regenerant to decrease conductivity. Calibration standards (1–500 µg/L) were formulated from a certified reference solution including Cl^-, F^-, Br^-, SO_4^{2-}, NO_3^-, and PO_4^{3-} (Thermo Fisher Scientific, USA). The total organic carbon (TOC) concentration of the samples was quantified using a Shimadzu TOC-L analyzer. Total nitrogen (TN) was assessed using a Hach DR 3900 spectrophotometer (Loveland, CO, USA) in conjunction with a TNT 828 kit.

Text S3: Detailed procedures for PFAS analysis

Adsorption experiment samples were centrifuged at 16,000 rpm for 15 minutes before PFAS measurement. Internal standards with isotope labels ($^{13}C_4$-PFOS and $^{13}C_2$-PFOA) were added before analysis in accordance with the steps outlined in EPA Method 537.1 (Revision 2.0). A 6470 Triple Quadrupole Mass Spectrometer (LC-MS/MS; Agilent Technologies, Santa Clara, CA, USA) was coupled with a 1290 Infinity II liquid chromatography system for PFAS measurement. The separation was accomplished using two Agilent Eclipse Plus C18 columns, one of which was a ZORBAX analytical column with dimensions of 3 × 50 mm and a particle size of 1.8 µm. The other column was a delay column with dimensions of 4.6 × 50 mm and a particle size of 3.5 µm. Both columns were kept at a temperature of 50 °C. Two solvents, A and B, a solution of 5 mM ammonium acetate in water and 95% methanol, respectively, made up the mobile phase. The ratio of A and B started from 70% A/30% B, changed to 0% A/100% B at 8 minutes and was maintained for 4 minutes before returning to the starting settings. The a flow rate was 0.5 mL/min. The overall run duration was 12 minutes.

Text S4: Adsorption kinetics, isotherms, and thermodynamic studies

Adsorption kinetic experiments were conducted using an initial PFAS concentration of 20 μg/L at 25 °C and 150 rpm, with an adsorbent dose of 100 mg/L and a pH of 6.8 for various contact times (1, 5, 15, 30, 60, 90, 120 min, and every 3 s within the first minute). The adsorption kinetics data were further analyzed and represented using three commonly used kinetic models: pseudo-first order (PFO), pseudo-second order (PSO), and intra-particle diffusion (IPD) [6]with these three equations:

PFO: $q_t = q_e(1 - e^{-k_1 t})$ (S1)

PSO: $q_t = k_2 q_e^2 t/(1 + k_2 q_e t)$ (S2)

IPD: $q_t = k_d t^{1/2} + C_d$ (S3)

The variables t (min), q_t (mg/g), and q_e (mg/g) denote the contact time, mass of adsorbate normalized by the mass of adsorbent at time t, and the equilibrium mass, respectively. Experimental findings determined the following: the rate constants for the PFO and PSO, k_1 (min^{-1}) and k_2 (g/(mg. min)), respectively; the IPD coefficient, k_d (mg/(g.$min^{1/2}$)); and C_d (mg/g) represents the constant associated with boundary layer thickness.

Various PFAS concentrations (10, 20, 200, and 500 μg/L) were used in adsorption isotherm tests at 25 ºC and 150 rpm for 4 h, with an adsorbent dose of 100 mg/L and a pH of 6.8. As seen in Eq. (4)-(6), three isotherm models — the Langmuir, Freundlich, and Sips — were employed to fit the adsorption data[7, 8]:

Langmuir: $q_e = K_L q_m C_e/(1 + K_L C_e)$ (S4)

Freundlich: $q_e = K_F C_e^{1/m}$ (S5)

Sips: $q_e = q_m (K_S C_e)^{1/n}/\left[1 + (K_S C_e)^{1/n}\right]$ (S6)

The equilibrium capacity, denoted by q_e (mg/g), is the ratio of the quantity of adsorbate (mg) and the adsorbent mass (g). Meanwhile, q_m (mg/g) denotes the theoretical adsorption capacity. The adsorbate concentration in the water phase at equilibrium is represented by C_e (μg/L). The Langmuir constant K_L certifies the adsorption capacity stated in L/g. Measured in units of mg·L^{-1}/m·(g·μg^{-1}/m), the Freundlich constant, K_F, denotes a substance's adsorption capability and energy. The Sips constant represents the adsorption affinity, which is shown as K_S (L/μg).

Adsorption thermodynamic studies were conducted following the same procedure as above, except at two additional temperatures (35 °C and 45 °C). Thermodynamic parameters such as free energy change (ΔG), entropy change (ΔS), and enthalpy change (ΔH) were calculated according to equations (S7-S8)[9]:

$$\triangle G^0 = \triangle H^0 - T\triangle S^0 \qquad \text{(S7)}$$

$$lnKc = \frac{-\triangle G^0}{RT} = \frac{\triangle S^0}{R} - \frac{\triangle H^0}{RT} \qquad \text{(S8)}$$

Where $\triangle G^0$ = the standard Gibbs free energy change, usually expressed in kJ mol^{-1}, $\triangle H^0$= the standard enthalpy change, reported in J mol^{-1}, $\triangle S^0$= the standard entropy change, reported in J. mol^{-1}K^{-1}, R = the universal gas constant, with a value of 8.314 J. mol^{-1}K^{-1}, and T is the absolute temperature, given in Kelvin (K).

Text S5. Mass calculation of PFAS added, removed by adsorption, and recovered by regeneration.

The mass balance of PFAS during adsorption and regeneration was quantified using a series of standard equations. The total mass of PFAS introduced into the system was calculated based on the initial concentration and volume of the test solution (Eq. S9). The amount of PFAS removed from the aqueous phase through adsorption onto the adsorbent was determined by the difference between the initial and residual concentrations at a given time point (Eq. S10). To evaluate regeneration efficiency, the mass of PFAS recovered from the adsorbent via methanol rinsing was measured (Eq. S11), and the percentage recovery was calculated as the ratio of recovered to sorbed mass (Eq. S12).

$$\text{Mass of PFAS added (ng)} = \frac{C_i}{1000} 50 \qquad \text{(S9)}$$

$$\text{Mass of PFAS removed (ng)} = \frac{C_i - C_t}{1000} 50 \qquad \text{(S10)}$$

$$\text{Mass of PFAS recovered from solvent rinse (ng)} = \frac{C_s \times V_s}{1000} \qquad \text{(S11)}$$

$$\text{Recovery of PFAS by regeneration (\%)} = \frac{\text{Eq. (S11)}}{\text{Eq. (S10)}} 100 \qquad \text{(S12)}$$

In these equations, C_i and C_t represent the initial and time-dependent concentrations of PFAS in the solution (ng/L), respectively; C_s is the PFAS concentration in the solvent rinse (ng/mL); V_s is the volume of the extraction solvent used (mL).

Table S1. Chemicals and reagents used in this study.

**Chemicals and reagents**	**Grade/purity**	**Supplier details**
Graphene nanoplatelets (1-5nm)	Analytical	ACS material
Ammonium hydroxide	28-30%	Fisher Scientific
Perfluorohexanoic acid (PFHxA)	≥ 98%	Frontier Scientific
Perfluoroheptanoic acid (PFHpA)	≥ 98%	Matrix Scientific
Perfluorooctanoic acid (PFOA)	≥ 96%	Sigma-Aldrich
Perfluorononanoic acid (PFNA)	≥ 98%	Oakwood Chemicals
Perfluorodecanoic acid (PFDA)	≥ 98%	Matrix Scientific
Potassium perfluorobutanesulfonate (PFBS)	≥ 98%	Accela Chembio Inc
Perfluorohexanesulfonic acid potassium salt (PFHxS)	≥ 98%	Frontier Scientific
Heptadecafluorooctanesulfonic acid potassium salt (PFOS)	≥ 98%	Sigma-Aldrich
Undecafluoro-2-methyl-3-oxahexanoic acid (GenX)	≥ 97%	SynQuest Laboratories
6:2 Fluorotelomer sulfonic acid (6:2 FTSA)	≥ 98%	SynQuest Laboratories
Perfluoro-n-[1,2-$^{13}C_2$]octanoic acid	≥ 98%	Wellington Laboratories Inc.
Sodium perfluoro-1[1,2,3,4-$^{13}C_4$]octanesulfonate	≥ 98%	Wellington Laboratories Inc.
Perfluoro-n-[1,2,3,4,6-$^{13}C_5$]hexanoic acid	≥ 98%	Wellington Laboratories Inc.
Cetyltrimethyl ammonium chloride	≥ 95%	Tokyo Chemical Industry
Ammonium acetate	LC/MS Grade	Fisher Scientific
Methanol	LC/MS Grade	Fisher Scientific
Ethanol	99.5 %	Fisher Scientific
Water	LC/MS Grade	Fisher Scientific

Table S2. The physicochemical properties of PFAS used in this study.

**Category**	**Compound name**	**Chemical structure**	**Chemical formula**	**Molecular weight (g/mol)**	**S_w (25 °C) (g/L)**	**pK_a (25 °C)**
Short-chain PFCA	Perfluorohexanoic acid (PFHxA)	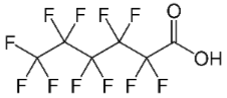	$C_6HF_{11}O_2$	314	15.7 [10]	-0.16 [11]
Short-chain PFCA	Perfluoroheptanoic acid (PFHpA)	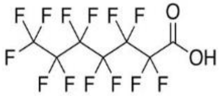	$C_7HF_{13}O_2$	364	3.65×10^{-3} [12]	−2.29 [12]
Long-chain PFCA	Perfluorooctanoic acid (PFOA)	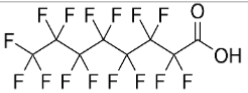	$C_8HF_{15}O_2$	414	3.4 [10]	-0.2 [11]
Long-chain PFCA	Perfluorononanoic acid (PFNA)	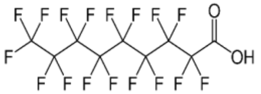	$C_9HF_{17}O_2$	464	6.25×10^{-2} [12]	-0.21[12]
Long-chain PFCA	Perfluorodecanoic acid (PFDA)	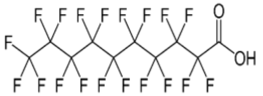	$C_{10}HF_{19}O_2$	514	9.5 [13]	-5.2 [14]
Short-chain PFSA	Potassium perfluorobutanesulfonate (PFBS)	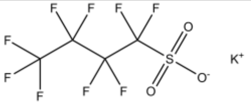	$C_4F_9O_3SK$	338	46.2 [15]	0.14 [11]

Long-chain PFSA	Perfluorohexanesulfonic acid potassium salt (PFHxS)	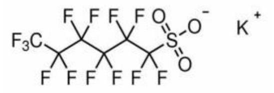	$C_6F_{13}KO_3SK$	438	2.3 [13]	0.14 [11]
Long-chain PFSA	Heptadecafluorooctanesulfonic acid potassium salt (PFOS)	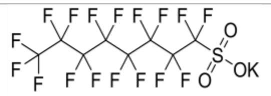	$C_8HF_{17}KO_3SK$	538	0.57 [10]	-3.27 [16]
PFOA alternative	Undecafluoro-2-methyl-3-oxahexanoic acid (GenX)	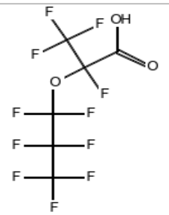	$C_6HF_{11}O_3$	330.05	N/A	2.84 [14]
PFOS alternative	6:2 fluorotelomer sulfonic acid (6:2 FTSA)	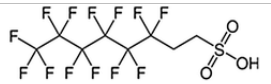	$C_8H_5F_{13}O_3S$	428	1.3 [13]	1.31 [13]

Note: Solubility in water (S_w); Dissociation constant ($_pK_a$); N/A: Data not available

Table S3. Dynamic multiple reaction monitoring transitions for the studied PFAS.

Compound name	Retention time (min)	Precursor ion	Quantification ion mass	Confirmation ion mass	Limit of detection (ng/L)
PFBS	3.6	299	99	80	2.4
PFHxS	5.7	399	99	80	1.2
PFHxA	4.67	313	269	119	2.0
PFHpA	5.63	363	319	169	4.1
GenX	4.9	285	185	169	4.1
PFOA	6.3	413	369	169	1.0
PFOS	6.84	499	99	80	1.7
PFNA	6.8	463	419	169	5.2
PFDA	7.24	513	469	218.7	2.1
6:2 FTSA	6.56	427	406.8	79.9	10.6
^{13}C-PFOA	6.3	415	370	N/A	N/A
^{13}C-PFOS	6.84	503	80	N/A	N/A

Note: N/A: data not available.

Table S4. Characteristics of the investigated Hudson River water.

**Parameter**	**Value**
pH	7.37 ± 0.01
TOC (mg/L)	3.38 ± 0.07
TN (mg/L)	1.00 ± 0.00
PO_4^{3-} (mg/L)	7.60 $\times 10^{-3}$ ± 0.00
Br^- (mg/L)	5.64 $\times 10^{-3}$ ± 0.00
Cl^- (mg/L)	BDL
F^- (mg/L)	BDL
NO_3^- (mg/L)	BDL
SO_4^{2-} (mg/L)	BDL
PFBS (ng/L)	14.64 ± 0.23
PFHxS (ng/L)	9.57 ± 0.83
PFHxA (ng/L)	25.50 ± 0.77
PFNA (ng/L)	15.50 ± 3.15
PFDA (ng/L)	11.68 ± 1.98
6:2 FTSA (ng/L)	5.86 ± 0.77

Note: TOC: total organic carbon; TN: total nitrogen; BDL: below detection limit.
Table adapted from Pervez et al. (2024) [17] licensed under CC-BY 4.0.

Table S5. Particle size, BET total surface area, and pore size distribution analysis.

**Adsorbent**	**Particle size (μm)**	**Surface area (m^2/g)**	**Total pore volume (cm^3/g)**	**Maximum pore width (Å)**
GNP	3.70	20.89	3×10^{-2}	197.26
GNP-CTAC	2.72	5.50	7.06×10^{-3}	205.60

Table S6. Adsorption performance comparison of the prepared GNP-CTAC with other similar adsorbents reported in the literature.

Adsorbent	Experimental conditions	PFAS studied	Equilibrium time	Isotherm	Adsorption capacity (mg/g)	References
rGO-ZF@CB	Adsorbent dose = 1000 mg/L, pH = 4, Conc. = 300 mg/L	PFOA, PFOS	120 min	Langmuir	16.07	[18]
Magnetic amino-functionalized graphene oxide	Adsorbent dose = 500 mg/L, pH = 6.5, Conc. = 0.5 mg/L	PFOA, PFOS, PFBS, PFHxS	30 min	N/A	N/A	[19]
$MgAl_2O_4$@CNTs	Adsorbent dose = 100 mg/L, pH = 7.5-9.0, Conc. = 0.1 mg/L	PFOA	270 min	Freundlich	175	[20]
Fe-doped graphitized biochar	Adsorbent dose = 1250 mg/L, pH = 6.5, Conc. = 0-100 mg/L	PFOA, PFBA	2880 min	Langmuir	PFOA = 38.6<br>PFBA = 10.1	[21]
Fluorine doped mesoporous carbon	Adsorbent dose = 1000 mg/L, pH = 6.6, Conc. = 1 mg/L	PFOS	7 days	N/A	0.99	[22]
Cu/CuO-CNTs	Adsorbent dose = 1000 mg/L, pH = 3, Conc. = 10-50 mg/L	PFOA	720 min	Freundlich	5	[23]
GNP-CTAC	Adsorbent dose = 100 mg/L, pH = 6.6, Conc. = 0.01 mg/L	PFBS, PFHxA, PFHxS, PFHpA, PFOA, PFOS, 6:2 FTSA, GenX, PFNA, PFDA	1 min	Sips	∑PFAS = 54.08	This study

Note: N/A: not available.

Table S7. Fitted isotherm modelling parameters for PFAS adsorption on GNP-CTAC.

Temperature (K)	Langmuir			Freundlich			Sips				Thermodynamic parameters		
	Q_{max} (mg/g)	K_L (L/μg)	R_L^2	K_F (mg·$L^{1/m}$/(g·$μg^{1/m}$))	m	R_F^2	q_m (mg/g)	K_S (L/μg)	n	R_S^2	△G (KJ.K/mol)	△H (KJ.K/mol)	△S (KJ.K/mol)
318	58.2434	0.0033	0.9992	1.4888	2.0155	0.9929	66.1946	0.0023	1.1564	0.9987	-4.3177	24.5346	0.0907
308	59.5566	0.0047	0.9734	1.5886	1.9612	0.9565	46.6185	0.0130	0.3905	0.9989	-3.4104		
298	51.4688	0.0294	0.9930	7.1359	3.2745	0.9721	54.0846	0.0248	1.2126	0.9980	-2.5031		

Table S8. Experimental and isotherm modeled values of total adsorbed PFAS at equilibrium (q_e) and concentrations in aqueous phase at equilibrium (C_e) in the adsorption process by GNP-CTAC at 25 °C.

**∑PFAS C_e (µg/L)**	**∑PFAS q_e experimental (mg/g)**	**∑PFAS q_e modeled (mg/g)**		
		**Langmuir**	**Freundlich**	**Sips**
0.38126475	1.13463179	0.570704683	5.315706297	1.13463179
0	1.753354704	0	0	0
0	4.893447375	0	0	0
25.105555	21.83420838	21.86069952	19.09521762	21.83420838
553.4050475	48.49482233	48.48951644	49.1072832	48.49482233

Table S9. Experimental and isotherm modeled values of total adsorbed PFAS at equilibrium (q_e) and concentrations in aqueous phase at equilibrium (C_e) in the adsorption process by GNP-CTAC at 35 °C.

**∑ PFAS C_e (µg/L)**	**∑ PFAS q_e experimental (mg/g)**	**∑ PFAS q_e modeled (mg/g)**		
		**Langmuir**	**Freundlich**	**Sips**
0.38126475	1.13463179	0.107445935	0.971619035	3.28988E-05
1.4204216	1.739150488	0.39833672	1.899893291	0.000954068
40.199721	4.491450165	9.532743845	10.44628856	4.493632367
89.717796	21.18808597	17.77132049	15.73008869	21.18746342
764.15952	46.3872776	46.67240345	46.88850509	46.38738707

Table S10. Experimental and isotherm modeled values of total adsorbed PFAS at equilibrium (q_e) and concentrations in aqueous phase at equilibrium (C_e) in the adsorption process by GNP-CTAC at 45 °C.

∑ PFAS C_e (μg/L)	∑ PFAS q_e experimental (mg/g)	∑ PFAS q_e modeled (mg/g)		
		Langmuir	Freundlich	Sips
0.38126475	1.13463179	0.074573844	0.922766968	0.155450622
0.8352907	1.745001797	0.163130753	1.36169637	0.305593343
23.4369595	4.65907778	4.254743575	7.120523894	5.067171196
163.061278	20.45465115	20.62582762	18.64140756	20.34199204
956.55708	44.463302	44.43014403	44.84223211	44.48164384

Table S11. Summary of binding energy and atomic concentration of GNP-CTAC.

Samples	Binding energy (eV)				
	C 1s	O 1s	N 1s	Cl 2p	F 1s
Before adsorption	284.6	532.5	401.3	198	
After adsorption	284.8	532.3	399.6		688.7
**Atomic concentration (%)**					
	C 1s	O 1s	N 1s	Cl 2p	F 1s
Before adsorption	79.28	19.57	0.69	0.46	
After adsorption	73.52	18.26	1.44		6.78

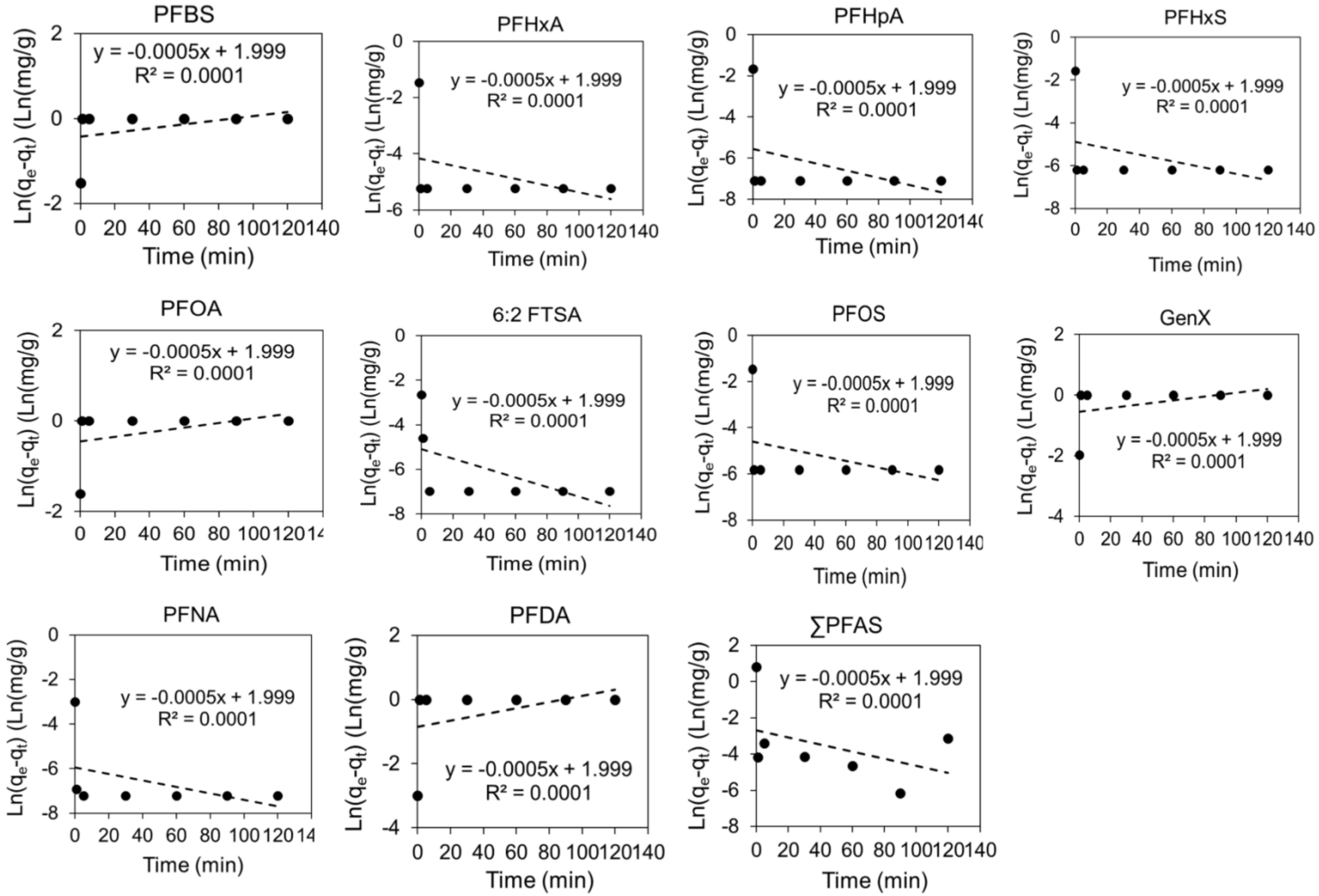

Fig. S1. Fitting of adsorption data of PFAS at the initial concentrations of 10 μg/L by the linear form of pseudo-first-order model.

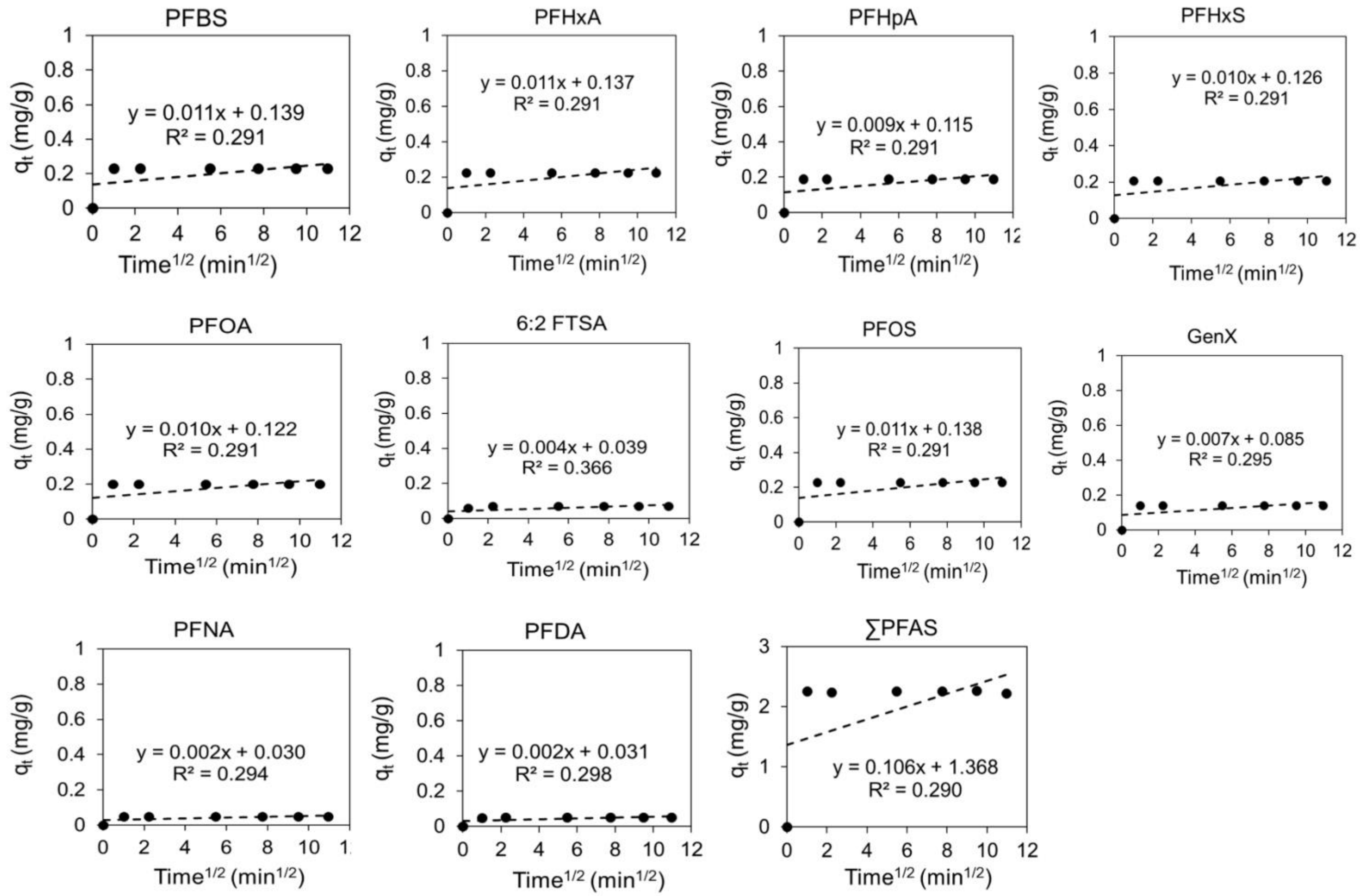

Fig. S2. Fitting of adsorption data of PFAS at the initial concentrations of 10 µg/L by the linear form of intra-particle diffusion model.

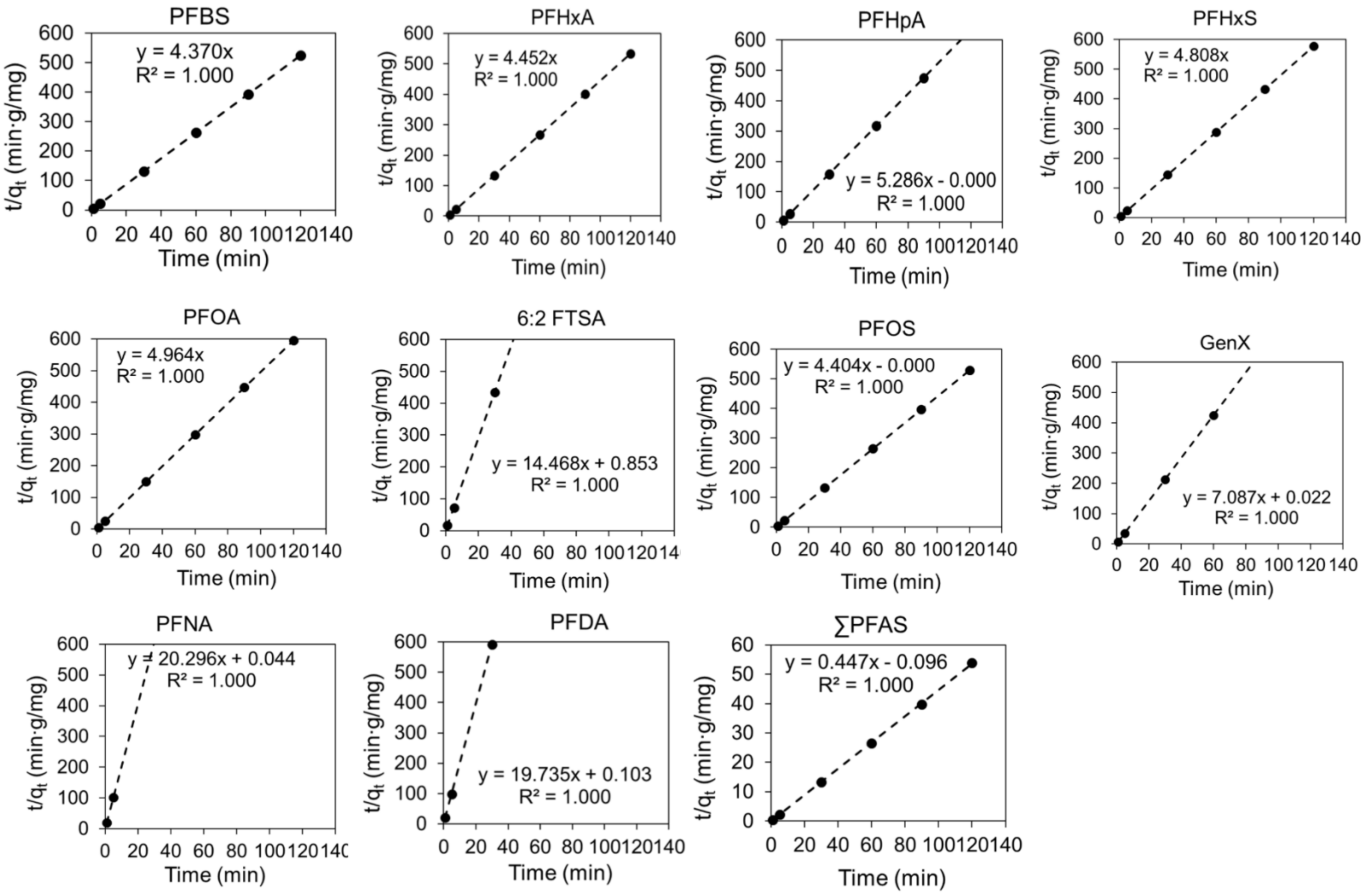

Fig. S3. Fitting of adsorption data of PFAS at the initial concentrations of 10 μg/L by the linear form of pseudo-second-order model.

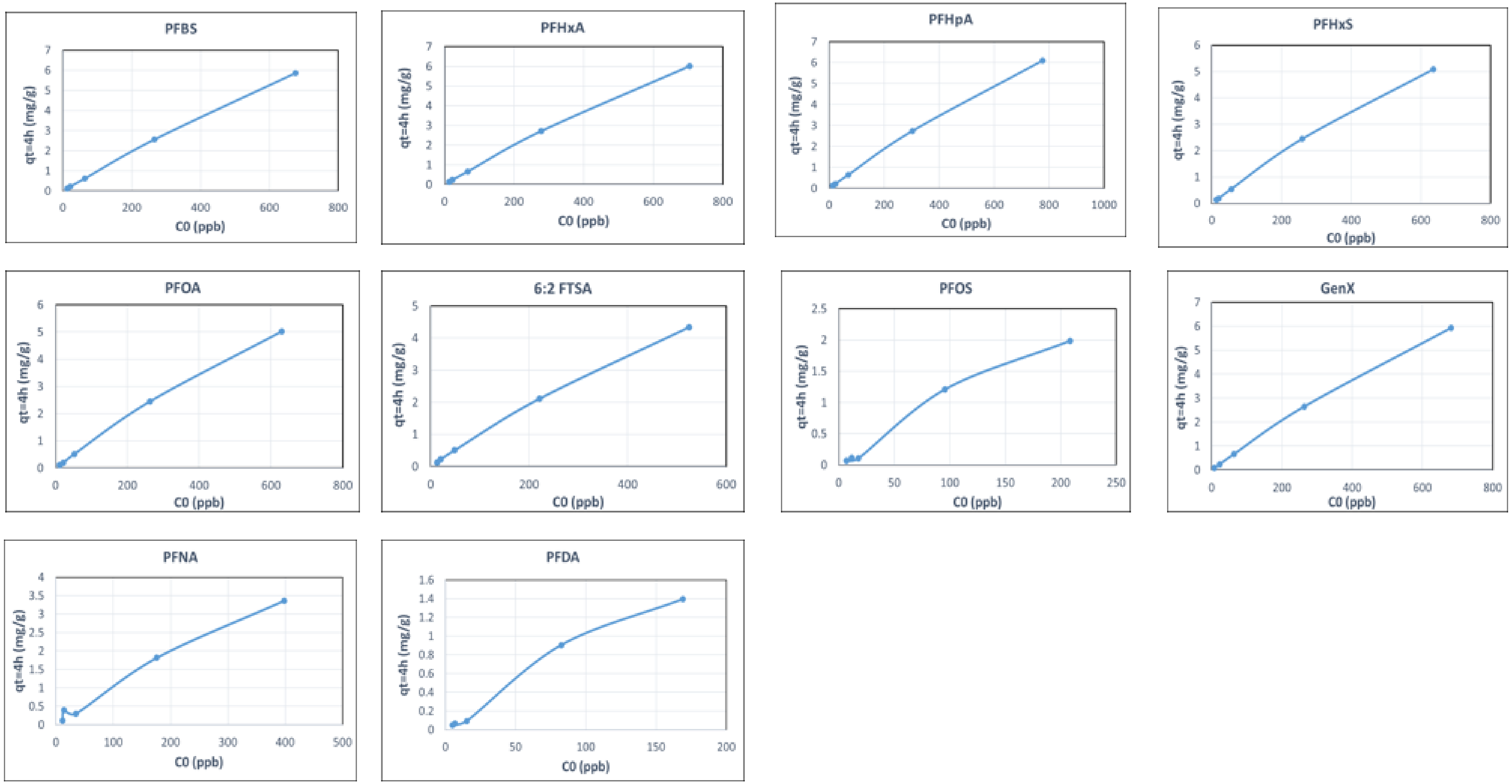

Fig. S4. The relationship between initial concentrations (C_0) and mass of PFAS adsorbed by the GNP-CTAC.

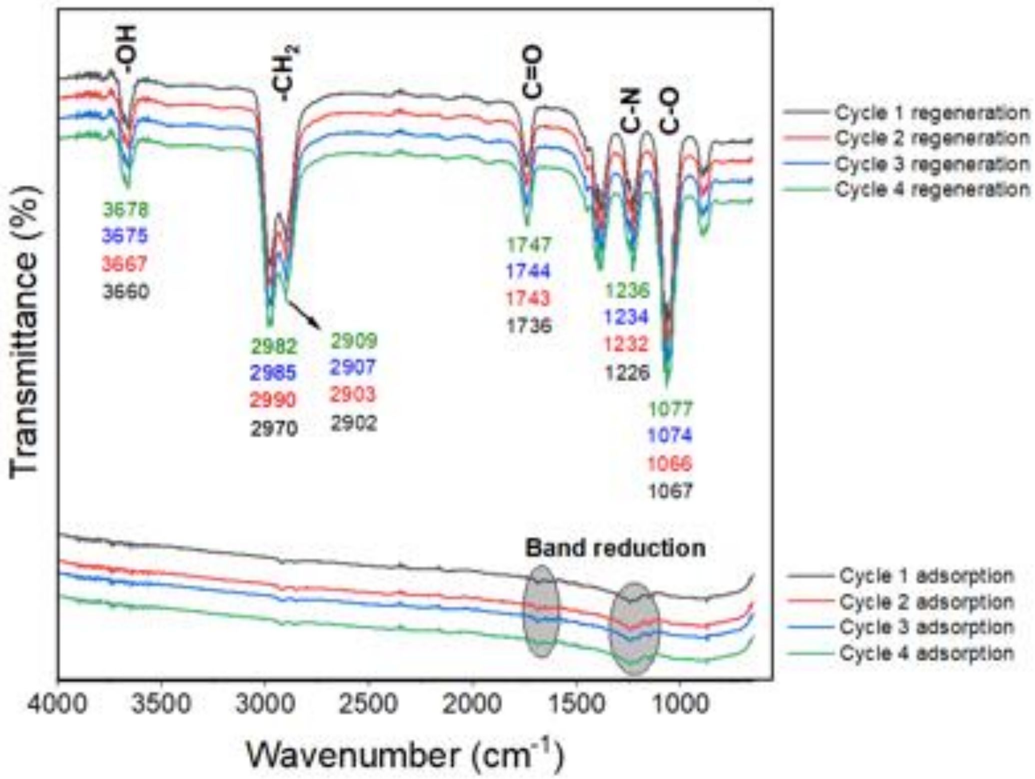

Fig. S5. FTIR spectra after four cycles of regeneration and reuse.

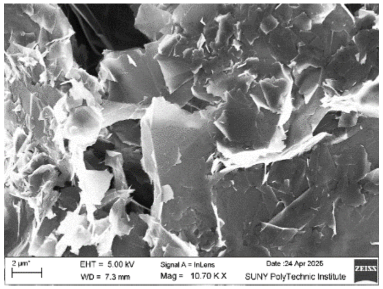

Cycle 1 adsorption

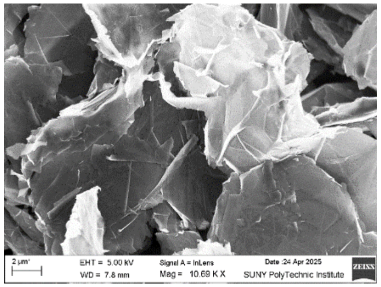

Cycle 2 adsorption

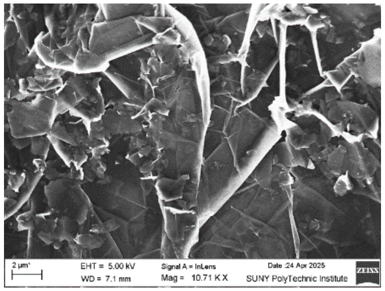

Cycle 3 adsorption

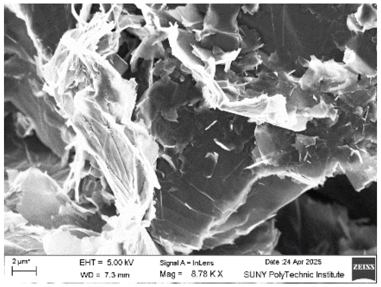

Cycle 4 adsorption

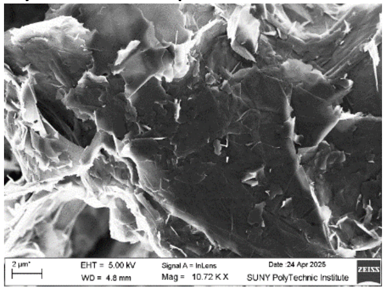

Cycle 1 regeneration

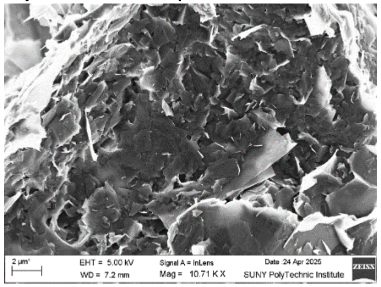

Cycle 2 regeneration

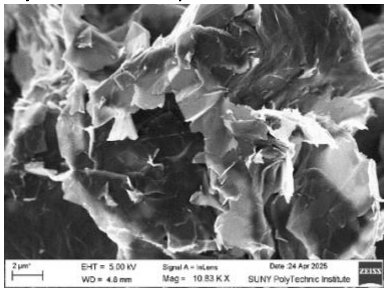

Cycle 3 regeneration

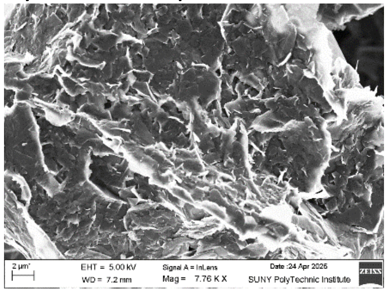

Cycle 4 regeneration

Fig. S6. SEM analysis during four adsorption-regeneration cycles.

References

(1) Zhang, W.; Zhang, Q.; Liang, Y. Ineffectiveness of ultrasound at low frequency for treating per- and polyfluoroalkyl substances in sewage sludge. *Chemosphere* **2022**, *286*, 131748.
(2) Zhang, W.; Liang, Y. Performance of different sorbents toward stabilizing per-and polyfluoroalkyl substances (PFAS) in soil. *Environmental Advances* **2022**, *8*, 100217.
(3) Zhang, W.; Jiang, T.; Liang, Y. Stabilization of per-and polyfluoroalkyl substances (PFAS) in sewage sludge using different sorbents. *Journal of Hazardous Materials Advances* **2022**, 100089.
(4) Jiang, T.; Zhang, W.; Liang, Y. Uptake of individual and mixed per-and polyfluoroalkyl substances (PFAS) by soybean and their effects on functional genes related to nitrification, denitrification, and nitrogen fixation. *Science of The Total Environment* **2022**, *838*, 156640.
(5) Jiang, T.; Pervez, M. N.; Quianes, M. M.; Zhang, W.; Naddeo, V.; Liang, Y. Effective stabilization of per-and polyfluoroalkyl substances (PFAS) precursors in wastewater treatment sludge by surfactant-modified clay. *Chemosphere* **2023**, *341*, 140081.
(6) López-Luna, J.; Ramírez-Montes, L. E.; Martinez-Vargas, S.; Martínez, A. I.; Mijangos-Ricardez, O. F.; González-Chávez, M. d. C. A.; Carrillo-González, R.; Solís-Domínguez, F. A.; Cuevas-Díaz, M. d. C.; Vázquez-Hipólito, V. Linear and nonlinear kinetic and isotherm adsorption models for arsenic removal by manganese ferrite nanoparticles. *SN Applied Sciences* **2019**, *1* (8), 950. DOI: 10.1007/s42452-019-0977-3.
(7) Ayawei, N.; Ebelegi, A. N.; Wankasi, D. Modelling and interpretation of adsorption isotherms. *Journal of chemistry* **2017**, *2017* (1), 3039817.
(8) Tzabar, N.; ter Brake, H. J. M. Adsorption isotherms and Sips models of nitrogen, methane, ethane, and propane on commercial activated carbons and polyvinylidene chloride. *Adsorption* **2016**, *22* (7), 901-914. DOI: 10.1007/s10450-016-9794-9.
(9) Aragaw, T. A. Utilizations of electro-coagulated sludge from wastewater treatment plant data as an adsorbent for direct red 28 dye removal. *Data in Brief* **2020**, *28*, 104848. DOI: https://doi.org/10.1016/j.dib.2019.104848.
(10) Fujii, S.; Polprasert, C.; Tanaka, S.; Hong Lien, N. P.; Qiu, Y. New POPs in the water environment: distribution, bioaccumulation and treatment of perfluorinated compounds–a review paper. *Journal of Water Supply: Research and Technology—AQUA* **2007**, *56* (5), 313-326.
(11) Steinle-Darling, E.; Reinhard, M. Nanofiltration for trace organic contaminant removal: structure, solution, and membrane fouling effects on the rejection of perfluorochemicals. *Environmental science & technology* **2008**, *42* (14), 5292-5297.
(12) Kim, S.; Chen, J.; Cheng, T.; Gindulyte, A.; He, J.; He, S.; Li, Q.; Shoemaker, B. A.; Thiessen, P. A.; Yu, B.; et al. PubChem in 2021: new data content and improved web interfaces. *Nucleic Acids Research* **2021**, *49* (D1), D1388-D1395. DOI: 10.1093/nar/gkaa971 (accessed 2/22/2023).
(13) Christensen, E. R.; Wang, Y.; Huo, J.; Li, A. Properties and fate and transport of persistent and mobile polar organic water pollutants: A review. *Journal of Environmental Chemical Engineering* **2022**, 107201.
(14) Pauletto, P. S.; Bandosz, T. J. Activated carbon versus metal-organic frameworks: A review of their PFAS adsorption performance. *Journal of Hazardous Materials* **2022**, *425*, 127810.
(15) Zhou, Q.; Deng, S.; Yu, Q.; Zhang, Q.; Yu, G.; Huang, J.; He, H. Sorption of perfluorooctane sulfonate on organo-montmorillonites. *Chemosphere* **2010**, *78* (6), 688-694. DOI: https://doi.org/10.1016/j.chemosphere.2009.12.005.

(16) Brooke, D.; Footitt, A.; Nwaogu, T. Environmental risk evaluation report: Perfluorooctanesulphonate (PFOS). **2004**.
(17) Pervez, M. N.; Jiang, T.; Mahato, J. K.; Ilango, A. K.; Kumaran, Y.; Zuo, Y.; Zhang, W.; Efstathiadis, H.; Feldblyum, J. I.; Yigit, M. V. Surface modification of graphene oxide for fast removal of per-and polyfluoroalkyl substances (PFAS) mixtures from river water. *ACS Es&t Water* **2024**, *4* (7), 2968-2980.
(18) Elanchezhiyan, S. S.; Preethi, J.; Rathinam, K.; Njaramba, L. K.; Park, C. M. Synthesis of magnetic chitosan biopolymeric spheres and their adsorption performances for PFOA and PFOS from aqueous environment. *Carbohydrate Polymers* **2021**, *267*, 118165. DOI: https://doi.org/10.1016/j.carbpol.2021.118165.
(19) Mahpishanian, S.; Zhou, M.; Foudazi, R. Magnetic amino-functionalized graphene oxide nanocomposite for PFAS removal from water. *Environmental Science: Advances* **2024**, *3* (12), 1698-1713, 10.1039/D4VA00171K. DOI: 10.1039/D4VA00171K.
(20) Yin, S.; López, J. F.; Solís, J. J. C.; Wong, M. S.; Villagrán, D. Enhanced adsorption of PFOA with nano MgAl2O4@CNTs: influence of pH and dosage, and environmental conditions. *Journal of Hazardous Materials Advances* **2023**, *9*, 100252. DOI: https://doi.org/10.1016/j.hazadv.2023.100252.
(21) Liu, Z.; Zhang, P.; Wei, Z.; Xiao, F.; Liu, S.; Guo, H.; Qu, C.; Xiong, J.; Sun, H.; Tan, W. Porous Fe-doped graphitized biochar: An innovative approach for co-removing per-/polyfluoroalkyl substances with different chain lengths from natural waters and wastewater. *Chemical Engineering Journal* **2023**, *476*, 146888. DOI: https://doi.org/10.1016/j.cej.2023.146888.
(22) Medha, S.; Romisher, Z.; Van Bramer, S.; Weyrich, J.; Khan, S.; Saha, D. Enhanced adsorption of perfluorooctanesulfonic acid (PFOS) in fluorine doped mesoporous carbon: Experiment and simulation. *Carbon* **2024**, *218*, 118745. DOI: https://doi.org/10.1016/j.carbon.2023.118745.
(23) Liu, L.; Li, D.; Li, C.; Ji, R.; Tian, X. Metal nanoparticles by doping carbon nanotubes improved the sorption of perfluorooctanoic acid. *Journal of Hazardous Materials* **2018**, *351*, 206-214. DOI: https://doi.org/10.1016/j.jhazmat.2018.03.001.
